# SOMSpec as a General Purpose Validated Self-Organising Map Tool for Rapid Protein Secondary Structure Prediction From Infrared Absorbance Data

**DOI:** 10.3389/fchem.2021.784625

**Published:** 2022-01-27

**Authors:** Marco Pinto Corujo, Adewale Olamoyesan, Anastasiia Tukova, Dale Ang, Erik Goormaghtigh, Jason Peterson, Victor Sharov, Nikola Chmel, Alison Rodger

**Affiliations:** ^1^ Department of Chemistry, University of Warwick, Coventry, United Kingdom; ^2^ Department of Molecular Sciences, Macquarie University, Sydney, NSW, Australia; ^3^ Center for Structural Biology and Bioinformatics, Laboratory for the Structure and Function of Biological Membranes, Campus Plaine, Université Libre de Bruxelles, Brussels, Belgium; ^4^ BioPharmaSpec Inc., Malvern, PA, United States

**Keywords:** protein, secondary structure, infrared absorbance, validation, self-organising map

## Abstract

A protein’s structure is the key to its function. As protein structure can vary with environment, it is important to be able to determine it over a wide range of concentrations, temperatures, formulation vehicles, and states. Robust reproducible validated methods are required for applications including batch-batch comparisons of biopharmaceutical products. Circular dichroism is widely used for this purpose, but an alternative is required for concentrations above 10 mg/mL or for solutions with chiral buffer components that absorb far UV light. Infrared (IR) protein absorbance spectra of the Amide I region (1,600–1700 cm^−1^) contain information about secondary structure and require higher concentrations than circular dichroism often with complementary spectral windows. In this paper, we consider a number of approaches to extract structural information from a protein infrared spectrum and determine their reliability for regulatory and research purpose. In particular, we compare direct and second derivative band-fitting with a self-organising map (SOM) approach applied to a number of different reference sets. The self-organising map (SOM) approach proved significantly more accurate than the band-fitting approaches for solution spectra. As there is no validated benchmark method available for infrared structure fitting, SOMSpec was implemented in a leave-one-out validation (LOOV) approach for solid-state transmission and thin-film attenuated total reflectance (ATR) reference sets. We then tested SOMSpec and the thin-film ATR reference set against 68 solution spectra and found the average prediction error for helix (α + 3_10_) and *β*-sheet was less than 6% for proteins with less than 40% helix. This is quantitatively better than other available approaches. The visual output format of SOMSpec aids identification of poor predictions. We also demonstrated how to convert aqueous ATR spectra to and from transmission spectra for structure fitting. Fourier self-deconvolution did not improve the average structure predictions.

## Introduction

Proteins are biomolecules with characteristic 3D shapes that determine their functions, e.g., structural, immune response, enzyme catalysis, and regulation (Lesk, 2010). In addition, there has been a growing interest in proteins as therapeutic agents over the past 20 years (Leurs et al., 2015). For a protein to be functional, it needs to be in a certain conformation; however, purification procedures often induce structural changes. To ensure the correct structure is retained/obtained during protein production and formulation, robust analysis methods must be used for regulatory as well as research purposes (Leurs et al., 2015).

Optical spectroscopic methods have the major advantage of not requiring a protein to form crystals, and they can be applied to any size molecules from peptide to high molecular weight assemblies. Circular dichroism (CD) spectroscopy is routinely used to estimate the secondary structure of unknown proteins and for batch-to-batch comparison of biopharmaceutical products (Woody, 1994; Sklepari et al., 2016; Spencer and Rodger, 2021). CD has the advantage of being relatively straightforward both to implement and to interpret. However, it has a number of limitations largely following from the need to keep the sample absorbance below a maximum of 2.5 at all wavelengths of interest and the need to know the concentration and path length. In aqueous solution, the protein concentration range is therefore practically limited to approximately 0.01—10 mg/ml protein. This is further restricted for biopharmaceuticals which are often formulated with high concentrations of non-protein absorbing components such as amino acids and chloride ions.

An alternative spectroscopic method to CD is mid-infrared absorption spectroscopy as the differential patterns in H-bonds and geometrical orientations of amide bonds in different secondary structure motifs affect the frequencies and intensities of vibrations. Protein IR spectra contain nine separate bands, referred to as Amide A, B, and I–VII (Kong and Yu, 2007; Rygula et al., 2013). It is generally accepted that the Amide I band (1,600–1700 cm^−1^) carries the most direct link to secondary structure content. Its vibrational contribution is from the C=O stretching of the amide group coupled with the in-phase bending of the N–H bond and stretching of the C–N bond (Krimm and Bandekar, 1986; Bandekar, 1992). Some side chains also absorb in the region; however, in this work, we ignore side-chain contributions because Venyaminov and Kalnin (Venyaminov and Kalnin, 1990) and Oberg (Oberg et al., 2004) found that subtracting side chain contributions provided only a moderate improvement to secondary structure determination. A great deal of work has been done on protein IR spectroscopy, but the best way to extract secondary structure information for regulatory or research purposes remains unclear.

The Amide I band is usually a featureless broad band so curve fitting methods, often preceded by band-narrowing, have been implemented to facilitate structure fitting (Kauppinen et al., 1981; Maddams and Tooke, 1982; Susi and Byler, 1983; Byler and Susi, 1986). Byler and Susi (Byler and Susi, 1986) developed a band-fitting method involving first a deconvolution procedure and then band-shape fitting with the Gaussian bands centred at the maximum (negative) values of the second derivative of the spectrum. They decided, after empirical analysis of over 20 proteins, that the relative areas under bands assigned to α-helix (∼1,654 cm^−1^), *β*-sheet (∼1,631 ± 7 cm^−1^ and ∼1,678 cm^−1^), and everything else corresponded to their relative secondary structure contents. (This has been assumed by other workers.) They found a fairly good match of their predictions with the Levitt and Greer’s algorithm for extracting secondary structure from crystal data (Levitt and Greer, 1977). However, Levitt and Greer noted in their original work that their approach significantly over-estimates β-structure, making Byler and Susi’s IR predictions a significant over-estimate of *β*-structure as deemed by other annotation approaches. The more recent consensus, e.g., (Kong and Yu, 2007; Yang et al., 2015), is that 1,620–1,640 cm^−1^ is attributed to *β*-sheet, 1,640–1,650 cm^−1^ to Other structures, 1,650–1,656 cm^−1^ to α-helix, and 1,670–1,685 cm^−1^ to turns. However, as noted by Oberg et al. (2004), band fitting usually requires a series of subjective decisions that can dramatically affect both result and interpretation. The authors arguably making the strongest claims for the efficacy of a band fitting approach (Yang et al., 2015) refer to their previous work on cytochrome-*c* (Dong et al., 1992) and to a paper by Kalnin et al. (1990). However, the Dong cytochrome-*c* result, while good for α-helix, has a 21–25% error in *β*-sheet content and Kalnin et al. (1990) used a reference set of proteins of known structure as their fitting approach rather than band fitting.

Various factor analysis methods have been applied to proteins using different reference sets. Lee et al. (1990), using a reference set of 18 protein IR spectra, concluded that they could predict protein secondary structure with standard errors of 4% for α-helix and 8% for β-sheet. Pancoska Keiderling and others used a reference set as well as principal component and factor analysis methodologies for both vibrational CD and IR spectra (Pancoska et al., 1991; Baumruk et al., 1996). Further refinement of the data through Fourier self-deconvolution did not improve their structure estimates (Wi et al., 1998). Dukor et al. and BioTools (Jupiter, US) have developed this approach into an easy-to-use fitting program by complementing the approach with their IR reference set and integrating it with data collection on their instrument. The resulting program *Prota* ^*TM*^ provides reasonably good structure estimates, but the details of the fittings cannot be interrogated by the user. Oberg et al. (2004) have extensively explored the application of a partial least squares analysis (PLS) with a 50-protein reference set and concluded that the most important factor is the quality of the reference set—it must cover the structure-space of interest.

Oberg et al. (2004) also considered application of the CD structure fitting program SELCON (Sreerama and Woody, 2000) to IR data which proved to give similar performance to the PLS analysis. They observed that larger reference sets usually do not perform better than smaller ones, as they may include more “anomalous” spectra—so it is important to be able to interrogate results rather than simply accept a number. Goormaghtigh et al. (2006) had significant success with an approach which identifies three key wavenumbers for the three structural features that can be distinguished in the IR spectrum. Their ascending stepwise method identifies the relevance of each wavenumber of the infrared spectrum for the prediction of a given secondary structure and yields a particularly simple method for computing the secondary structure content. The original work has been successfully extended to high throughput secondary structure determination by collecting data in an array format (De Meutter and Goormaghtigh, 2021). However, the preference for a data point in the Amide II band is a concern for biopharmaceutical samples as we have observed that the magnitude of this band varies significantly with formulation vehicle. A different choice of optimal wavenumbers could make the analysis more universal.

Our experience of using CD for the analysis of biopharmaceutical protein structure has convinced us that the most important aspect of a structure fitting approach is to know its limitations. Extensive work has been done to validate methods that determine structure from CD spectra of unknowns. The most widely used methods for CD analysis, e.g., CDsstr (Johnson, 1988) and SELCON3 (Sreerama and Woody, 2000), all use a reference set of spectra of proteins of known secondary structures. Our self-organising map approach (Hall et al., 2014a; Hall et al., 2014b) uses a different approach from CDsstr and SELCON, but we have shown it is equally reliable and it has the advantage that it produces output that enables the user to interrogate what is behind secondary structure estimates. When we needed to develop robust methods for analyzing protein infrared absorbance spectra, we therefore adapted our self-organising map analysis, now called SOMSpec, to be used for structure fitting from Amide I IR spectra (Corujo et al., 2018) and found it seemed to work well for the few examples we considered, though it depended on the reference set of spectra and structure assignments. SOMSpec is described in the Materials and Methods section and the Supplementary Material. The goal of this work was to develop an easy-to-use protein IR spectra analysis platform based on the SOMSpec program and to determine how well it works for various datasets of transmission, Fourier self-deconvolved spectra, and attenuated total reflectance (ATR) spectra. We also provide the means to transform ATR spectra into transmission for slightly improved secondary structure predictions against a transmission reference set. The endpoint of the work is a clear idea of how reliable SOMSpec is for this application and where the user must interrogate the output for further information.

## Materials and Methods

### Secondary Structure Annotation

The hydrogen-bonding pattern-based Dictionary of Secondary Structure of Proteins (DSSP) which divides protein secondary structure into 8 major classes abbreviated as follows: 3_10_-helix (G), *α*-helix (H), *π*-helix (I), *β*-sheet (E), *β*-bridge (B), turn (T), bend (S), and coil (C) is used in this work. The different reference sets combine the categories differently to reduce the number of classes (Wi et al., 1998). Annotations may be found in http://2struc.cryst.bbk.ac.uk [(Whitmore and Wallace, 2004) and (Oberg et al., 2003)]. Based on the results of reference (Spencer and Rodger, 2021) for CD spectroscopy and reference (Oberg et al., 2004) for IR, we limited our final discussions to three categories which we refer to as α-helix or helix (which includes *α*-helix and 3_10_-helix), *β*-sheet, and other (which is the combination of the rest of secondary structure types). As any residue belongs either to the helix or sheet or Other category, we only explicitly present the helix and sheet results in our figures. The deviations for Other are simply minus the sum of the helix and sheet deviations.

### Self-Organising Map Spectral Fitting

#### SOMSpec Operates in Three Steps

i) *Training the map*: in the first step, an unsupervised self-organising (or Kohonen) map approach creates a 2D square array and organises the reference set protein IR spectra to cluster them in terms of spectral similarity, with the similarity being represented by distances. Each node of the map has a spectrum allocated to it. Unless a reference spectrum sits on a node, a distance-weighted mean of neighbouring reference spectra is assigned to each node. A trained map can be used repeatedly as long as the wavenumber range of the test spectrum is the same as the reference spectra. For the leave-one-out validations (LOOV, see below), we trained for 20,000 steps and for full reference sets for 50,000 steps. The optimal map dimension (Hall et al., 2013) is somewhat lower than the reference set size, so we used 20 × 20 for the solid-state reference set and 40 × 40 for the film one.
ii) *Structure assignment*: in the second step, a vector which summarises the secondary structure of the spectrum assigned to a node is also assigned to the node. Reference spectra nodes take the reference spectra secondary structure vector. Nodes with distance-weighted sum spectra have secondary structure assigned in the same way.
iii) *Test*: unknown spectra are tested against the map by identifying nodes that are the best matching units (BMU) for the unknowns in terms of the distance in the spectral space. The secondary structure of the test spectrum is determined by a distance-weighted average of secondary structure of the top 5 or 3 best matching nodes or units (BMUs) in terms of the Euclidean distance on the map.

SOMSpec input files are created as comma-separated txt files. For an *N-*member reference set, the training file consist of *N* vertical columns of spectral data, separated by commas, with the corresponding structural data placed below. The test files are in the same format but without the structural information. The files were either created manually using Excel (*via* the basic .csv output format then renamed with the. txt extension) or automatically produced by a MATLab™ code.

SOMSpec output includes Normalised Root Mean Square Deviations (NRMSD, see Supplementary Materials for details) between experimental and predicted spectra, a plot of the trained map and the overlay of experimental and predicted spectra, the secondary structure predictions, and all the files to enable the plots to be regenerated.

More details about SOMSpec are given in the Supplementary Material which also contains a summary of the input and output information used below. The SOMSpec App [coded in MATLab™ (MathWorks, Chatswood, Australia)] and example input and output files may be found in the data repository which can be accessed via the Supplementary Material.

### Leave-One-Out Validation

In LOOV testing the spectra and secondary structure assignments of *N*–1 proteins out of *N* proteins in an IR reference set are used as the training set to generate a SOM. Then, the *N*th spectrum is tested against that SOM. This is repeated *N* times. The SOMSpec LOOV training files consist of *N*–1 vertical columns of spectral data, separated by commas, with the corresponding structural data placed below. The test files for the LOOV are a single column of spectral data. The results of the *N* LOOV tests give an indication of performance of the method−reference set combination.

### Spectra Transformations

*ATR to transmission and transmission to ATR:* We used the methodology developed in reference (Rodger et al., 2020) to convert ATR spectra into transmission spectra and inverted the methodology to convert transmission spectra into what would be collected on the same sample with a 45° incidence zinc selenide (ZnSe) ATR crystal. In summary (see Supplementary Material for more details), the relationships between ATR and transmission spectra we used are

$$A_{\mathrm{protein}}^{\mathrm{ATR}}={\left(\mathrm{\varepsilon C}\right)}_{\mathrm{protein}}\left(ad_{p}f\right)\left(1-\left(\ln10d_{p}f\right){\left(\mathrm{\varepsilon C}\right)}_{\mathrm{water}}\right)$$

$$A_{\mathrm{protein}}^{\mathrm{Transmission}}=\frac{A_{\mathrm{protein}}^{\mathrm{ATR}}\ell }{\left(\left(d_{p}f\right)\left(1-\left(\ln10d_{p}f\right){\left(\mathrm{\varepsilon C}\right)}_{\mathrm{water}}\right)\right)}$$

where $A_{protein}^{ATR}$ denotes the protein’s ATR absorbance, $d_{p}f$ is the penetration depth times the light intensity factor and εC=A/ℓ is the protein’s extinction coefficient times its concentration, where ℓ is the transmission path length. As the protein absorbance is much smaller than that of water in our experiments, we used $d_{p}f$ for water. Given the above equation is linear in ${\left(\varepsilon C\right)}_{protein}$ , we do not need to know the protein concentration or extinction coefficient or path length if we ultimately plan to normalise the spectra for structure fitting.

*Fourier self-deconvolution (FSD)*: OriginPro 2021 (Originlab, 2021) was used to perform FSD with the gamma and smoothing factor parameters varied. Each deconvolved spectrum was re-zeroed and re-normalised to 1 at its maximum value.

### Band Fitting

Two different band fitting approaches were undertaken. The first uses peak deconvolution within Origin Pro (Originlab, 2021) to fit the absorption bands directly to Gaussians after baselining the Amide 1 band by drawing a straight line from 1,600 cm^−1^–1700 cm^−1^. The secondary structure was identified by normalising the total area under the bands to 1 and then summing the areas of the bands that occurred in the accepted wavelength regions for each type of secondary structure: 1,620–1,640 cm^−1^ for *β*-sheet and 1,650–1,656 cm^−1^ for α-helix. If the answers were obviously wrong, the wavelength range was expanded slightly to favour the band-fitting approach. The approach outlined in (Yang et al., 2015) which involves taking the second derivative then band-fitting was also attempted. OriginPro 2021 (Originlab, 2021) was used to take second derivatives and to perform band fitting to Gaussians of minus the second derivative spectrum. The OriginPro fitting methodologies used in this work are detailed in the Supplementary Material, and the OriginPro files are provided in the data repository.

### Materials and Data Collection

All the proteins used in this work were purchased from Sigma Aldrich (Poole, United Kingdom) or available in-house. Using 2 different Jasco J-4200 (Jasco, Hachioji, Japan) spectrometers, spectra were collected with 64–1,000 scans with 4 cm^−1^ resolution, cosine apodization, and wavenumber range from 400 to 4,000 cm^−1^. The instruments were flushed with nitrogen (N_2_) at ∼30 L/min flow rate for 10 min to stabilise the water vapour contribution. The sample chamber flow rate was decreased to 5 L min^−1^ during data collection, and the interferometer was closed to the nitrogen flow. We collected 30 solid-state transmission spectra (see below), 19 aqueous transmission spectra (see below), and 2 aqueous ATR spectra. Baseline water spectra (18.2 MΩ Milli-Q water) were subtracted from the aqueous protein spectra to produce a flat line in the 2,100 cm^−1^ libration band region. A small scaling factor was sometimes required. If the spectrum could not be made flat in that region, the data were discarded. The integrated absorbance of the 1717–1772 cm^−1^ or 3,800–3,900 cm^−1^ regions were used to guide water vapour subtraction where necessary (Max and Chapados, 2009). A vapour spectrum was collected by first purging the instrument with N_2_, collecting a spectrum, then stopping the N_2_ flow (which allows a small increase in water vapour in the light beam) and collecting a second spectrum. The difference between the two spectra was used for water vapour correction. In practice, the need for water vapour correction was minimised by collecting a baseline water spectrum directly before each protein spectrum—so both spectra had similar water vapour contributions. Protein spectra were normalised to 1 at the Amide I maximum.

Solid-state data were collected using samples prepared by grinding proteins to a fine powder before mixing with separately grounded potassium bromide (KBr) to obtain a 1–10% w/w dilution of the protein. The KBr/protein mixture was compressed by means of a Manual Hydraulic Press (Specac, Orpington, UK) using 5–10 kpsi for about a minute to produce a pellet which was held between sodium chloride (NaCl) windows in a PIKE Technologies cell (Fitchburg, United States). Since liquid water absorption was detected, a scaled water spectrum collected separately with calcium fluoride (CaF_2_) windows was subtracted to give a flat spectrum in the 2,100 cm^−1^ region (Max and Chapados, 2009).

Aqueous protein solutions were prepared by dissolving lyophilised protein powders in 18.2 MΩ *cm* Milli-Q water in concentrations ranging from 10 to 80 mg ml^−1^. Insoluble residues were removed by centrifugal filtration with Teflon disk filters (0.22 µm pore size). Solution transmission spectra were collected using a Specac (Orpington, United Kingdom) transmission cell with CaF_2_ windows and no spacer making an estimated 1 µm path length. About 40 µl of sample was placed on one of the windows and the other was slid over it, making sure no air bubbles got trapped in the process. Two high *β*-sheet aqueous proteins samples were collected in ATR mode using a Pike Miracle™ ATR unit.

In addition, a 50-protein reference set previously obtained using ATR with thin films that were made by slowly evapourating aqueous protein solutions containing 100 µg of protein under a stream of N_2_ (Goormaghtigh et al., 2006) was used as the main reference set for this work. The proteins in the 50-protein thin-film set were selected to cover structure and fold space (Oberg et al., 2003; Goormaghtigh et al., 2006). A 47-spectra normalised aqueous transmission reference set provided by BioTools (Jupiter, USA) was used as an additional test set.

## Results

The goal of this work was to determine how we could optimise and validate the accuracy and reliability of secondary structure predictions for proteins from good quality protein IR absorbance spectra. A key goal was to have a procedure that required no intelligent intervention until the final analysis of the results. For validation of protein secondary structure fitting methods, the key questions to be answered are:

i) when can the fitting be trusted (most relevant for day-to-day applications)
ii) if the fitting is poor, why? (most relevant for method assessment).

What is presented here is the largest consideration of protein IR data that has been performed to date. We have worked with the reference-set based method SOMSpec, which we designed for CD, to extract structure information for an unknown protein spectrum by finding combinations of known proteins that most resemble the unknown using a self-organising map. We undertook a leave-one-out validation within a large reference set and then tested against a larger set of unknowns from different sources. We also considered to what extent the band shape enhancement of FSD facilitates how SOMSpec extracts information from the broad largely featureless bands of protein IR spectra. The structure information content of solid-state IR spectra, which are even broader than aqueous spectra, is assessed, as is whether transmission and ATR Amide I spectra can be compared. We also perform direct and second derivative band-fitting estimates on the same reference set in order to be able to compare the performance of the approaches most commonly used in the literature relative to an approach using the information in a reference set.

### Film Protein IR Spectra LOOV

**FIGURE 1:**
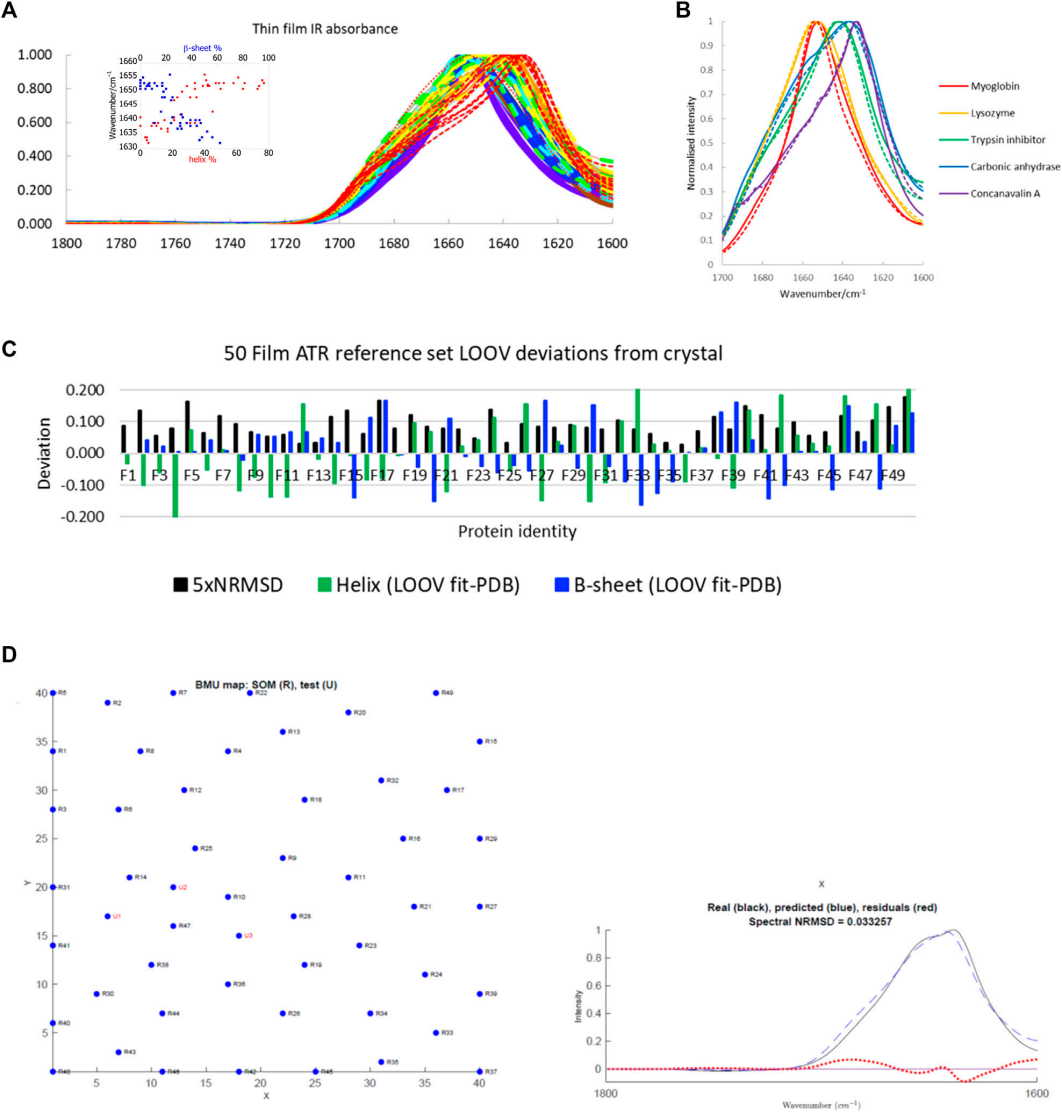
**(A)** A 50-protein thin-film ATR reference set (see Supplementary Table S1 for list of proteins). Inset: Amide I maxima plotted versus total *α*-helix red and *β*-sheet blue content. Proteins F1−F7 (>60% helix) are purple; F8−14 (45–59% helix) are blue; F15−21 (34–44% helix) are turquoise; F22−F28 (26–33% helix) are green; F29−F33, F36, F38 (17–25% helix) are yellow; F34, F35, F37, F39, F40 (10–16% helix) are orange; F41−F50 (<10% helix) are red with the unfolded F50 dotted. **(B)** Overlay of some normalised ATR thin-film (solid lines) and aqueous transmission (dashed lines) spectra. **(C)** LOOV deviations of SS prediction from PDB structures for helix (*α*-helix + 3_10_-helix) and *β*-sheet for the Amide I 50-protein thin-film reference set in order of decreasing helix content from left to right. 5 × NRMSD of the spectral fit is overlaid. Other category deviations are minus the sum of helix and *β*-sheet deviations. **(D)** Phosphoglycerate kinase (F17) LOOV SOMSpec output from 50-protein film reference set for a relatively poor quality example. In the map, U1, U2, U3 are the best matching nodes for the test protein. These can be expressed as linear combinations of their neighbouring reference set nodes. The proteins can be identified from Supplementary Table S1 in the SM, by noting that the test protein is F17 in the reference set, so proteins R1–R16 correspond to F1–F16, and R17–R49 correspond to F18–F50. The *real* spectrum is F17’s input data, and the *predicted* spectrum is the SOMSpec output.

The main protein IR reference set used in this work is a large one available in the literature (Figure 1A, see Supplementary Table S1 in the Supplementary Material for list of proteins and spreadsheets with SOMSpec input and output data). It is a 50-protein film reference set designed to give structure and fold coverage (Oberg et al., 2003; Goormaghtigh et al., 2006). The data for 50 proteins were collected by drying aqueous protein samples on an ATR unit to a thin film (we refer to this as the 50-protein thin film reference set). This approach has the advantage that the water absorbance of the spectra, which needs to be removed to give the protein contribution, is small rather than dominating the signal. However, it raises the question whether the spectra are an appropriate reference set for aqueous spectra. We expected the ATR film spectra to have the same spectral shape as transmission spectra based on reference (Jang and Miller, 1993) (if the proteins are folded the same). However, we were concerned that the film spectra might be less structured than solution spectra as is observed for solid-state data (see below). Figure 1B contains the overlay of some film and solution spectra for a few proteins of different secondary structure content. The spectra differ no more than independently collected aqueous transmission spectra vary which gave us the confidence to use this reference set as the main training set for SOMSpec IR in this work.

The correlation between intensity maximum position and helix or sheet content for the 50-protein thin film reference set is illustrated in the Figure 1A inset. On a simple level, there is a correlation between peak position and low α-helix/high *β*-sheet content, which is the basis for the band-fitting approaches. Peak position enables high, medium, and low α-helical and *β*-sheet proteins to be directly identified.

To test the performance of SOMSpec with proteins whose data were collected in an entirely consistent manner, LOOV analysis was performed. The deviations of the predicted fractions of α-helix and *β*-sheet from the Protein data bank (PDB) (Berman et al., 2000) DSSP are summarised in Figure 1C where the difference between the SOMSpec prediction and the DSSP annotation is plotted for helix (α + 3_10_) and *β*-sheet. The deviation for Other structures is minus the sum of these two (as both prediction and DSSP content sum to 1). The LOOV average helix (α + 3_10_) prediction error is 8% and the average *β*-sheet error is 7% (when the unfolded metallothionein II (F50) is excluded).

Figure 1D shows the LOOV graphical output for phosphoglycerate kinase (protein F17). The top graph illustrates the trained SOM with the BMUs for the test protein overlaid. Although there are 1,600 nodes in the map, only those corresponding to the 49 LOOV training set proteins are shown as blue dots with black labels. In this case, the fit is poor as shown by the BMUs not clustering (which indicates the test spectrum does not resemble spectra in any area of the map), a high spectral NRMSD, and poor maximum intensity wavenumber match. If the training is repeated, the map may look different with nodes moved, but the BMUs and structure predictions are almost the same because the nodes’ relationships are regenerated.

For practical application of any fitting method to unknown spectra, an estimate of the error for that specific sample is needed. The spectral NRMSD (5×NRMSD is plotted in Figure 1C to aid visualisation) gives an indication of how well the test spectrum overlays the best spectrum generated from the combination of spectra from the other *N*−1 spectra in the reference set. This together with the accuracy of the predicted *versus* experimental wavenumber maximum are guides to fit-quality. To get a more detailed picture of the reliability of SOMSpec, all helix and sheet errors above 10% were individually analysed. Caveats to emerge are:

i) Poor water or water vapour correction causes problems (e.g., F48). If this was a test spectrum, the data should be discarded. As it is part of a published reference set we retained it.
ii) Metallo-proteins whose ligand IR signals contribute to the Amide I region of the spectrum cause secondary structure prediction errors both for their own analysis and where they are BMUs (e.g., F10, F12, F26).
iii) 77% helix F4 (haemoglobin) and 41% helix F12 (cytochrome c) have almost identical spectra so any fit involving either of these as a BMU can only be concluded to have helix >40%.
iv) Predicted and original spectra that have a poor match of a high wavenumber maxima >1,650 cm^−1^ and/or miss significant high wavenumber intensity indicate helix secondary structure errors.
v) Predicted and original spectra that have a poor match of a low wavenumber maxima (<1,645 cm^−1^) and/or miss significant low wavenumber intensity indicate *β*-sheet secondary structure errors.
vi) Immunoglobulins (F42) only give good fits when an immunoglobulin is present in the reference set.
vii) F46 and F50 both have ∼70% random structure which is under-estimated by ∼40% and causes problems when they are BMUs.

The poor protein fits in the LOOV for the 50-protein film reference set are annotated in the final column of Supplementary Table S1.

### Aqueous Test Spectra of Known Structure

**FIGURE 2:**
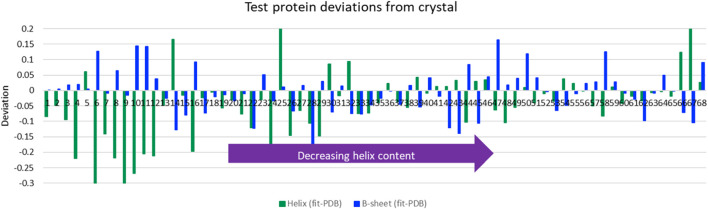
Deviations of predictions from PDB structures for average helix (*α*-helix + 3_10_-helix) and β-sheet for Amide I of 68 aqueous test proteins presented in order of decreasing helix content from left to right. Other category deviations are minus the sum of helix and *β*-sheet deviations.

The LOOV results gave guidance for the use of SOMSpec with unknowns. We therefore tested SOMSpec on a further 68 transmission spectra of various proteins with known secondary structure content using a SOMSpec map trained for the 50-protein thin-film reference set (the trained map is available *via* the Supplementary Material). Nineteen of the test spectra were collected in transmission mode, 2 were collected by ATR and transformed computationally to transmission (see methods and Supplementary Material spreadsheet), and 47 were from the commercial BioTools reference set. The average total helix and sheet errors were 8 and 5%, respectively (see Figure 2 for deviations from PDB structures), with the major contributions to the helix error being for high helix content proteins. When only the results for proteins of helix content <48% are considered, the helix and sheet average errors are both 5–6%, leading us to conclude that high helix proteins contribute disproportionately to the absolute helix errors. In general, the SOMSpec output plots present warnings where needed as listed above.

### Fourier Self-Deconvolution

It is widely accepted for IR spectroscopy applications that FSD can improve analysis. This is partly visual, which is important in most band fitting approaches, but may perhaps also be because it can remove noise from a spectrum. The effects of different parameters are illustrated in the Supplementary Material (Supplementary Figure S1) for bovine serum albumin. LOOV testing of the BioTools 47-protein reference set with a range of FSD parameters made significant improvements in the spectral NRMSDs, e.g., γ = 25 and smoothing factor = 0.5 together with re-zeroing and re-normalising improved average spectral NRMSDs by ∼30%; however, disconcertingly, the average error of structure predictions from PDB structures for these parameters increased marginally (1–2%). Less dramatic parameters, e.g., γ = 10, smoothing factor = 0.25, showed a marginal average improvement in secondary structure estimates, though this probably correlates with the noise reduction of the FSD process.

### Fitting ATR Spectra With a Transmission Reference Set

Because we often wish to study proteins in their native environment and aqueous ATR experiments are much easier to perform than transmission, we also investigated the quality of the secondary structure predictions for aqueous ATR spectra. Due to the instrument to instrument and sample to sample (e.g., concentration and buffer components) differences of ATR spectra, we decided that we should use a reference set that was instrument independent (in this case, the 50-protein thin film reference set). We produced ATR test spectra by transforming our transmission spectra for 21 aqueous test proteins to ATR following the equations given in the Supplementary Material which are based on reference (Rodger et al., 2020) (assuming a single bounce 45° incidence ZnSe crystal). The average SOMSpec helix prediction for the ATR spectra was somewhat worse than the corresponding transmission tests at 8 versus 6% for this subset of proteins, but the sheet prediction was marginally (1%) better. Again, visual inspection of output made problems obvious.

### Solid-State Protein IR Spectra LOOV

**FIGURE 3:**
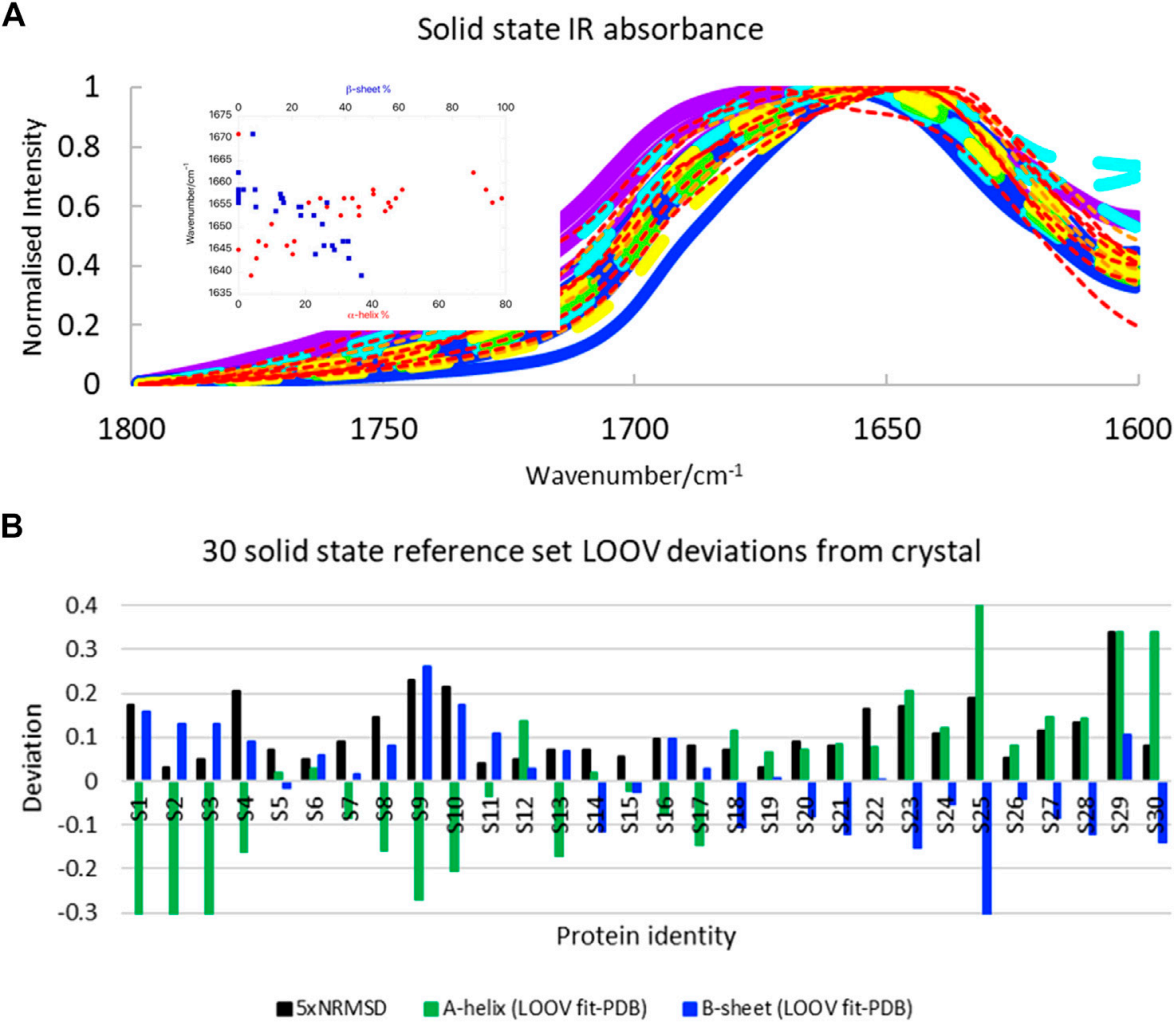
**(A)** Amide I transmission IR spectra of 30 solid-state proteins normalised to 1. Proteins with α-helix content >45% are indicated with broad lines and have maxima above 1,650 cm^−1^. Papain and lysozyme are broad dashed lines (see text). Colour coding of spectra is the same as in Figure 1: >60% helix purple; 45–59% helix blue; 34–44% helix turquoise; 26–33% helix green; 17–25% helix yellow; 10–16% helix orange; <10% helix red. Inset: Amide I maxima plotted against *α*-helix and *β*-sheet content. **(B)** LOOV deviations of SS prediction from PDB structures for *α*-helix and *β*-sheet for the Amide I of 30-protein solid-state reference set presented in order of decreasing helix content from left to right (for protein identities see Supplementary Material). Other category deviations are minus the sum of helix and *β*-sheet deviations.

We were interested to test how well SOMSpec worked with Amide I solid-state data, since solid-state proteins are more likely to have the same structures as those used for crystallography and the protein absorbance is not dominated by the water signal. The solid-state spectra (Figure 3A, see Supplementary Material for list of proteins and spreadsheets with SOMSpec input and output data) are broader and less structured than the 50-protein film reference set (Figure 1A). The correlation between position of the intensity maximum and helix or sheet content is slightly worse for the solid-state protein set than for the 50-protein film reference set.

The SOMSpec LOOV results for the 30-protein solid-state reference set are summarised in Figure 3B (see Supplementary Material for input and output details) in terms of deviations of α-helix and *β*-sheet content from PDB values. The NRMSD levels (plotted as 5×NRMSD) are generally a guide to the quality of the fit. Overall, SOMSpec gave reasonable estimates of secondary structure content with a few notable exceptions. The average deviations are 17% for α-helix and 10% for β-sheet. If we remove three types of problematic proteins from the average error calculation the average errors reduce to 11 and 8% respectively. The problem classes are again 1) high helix content proteins especially those with papain (S19) and/or lysozyme (S12) among their BMUs (the helix content is significantly underestimated), 2) β_II_ proteins in particular trypsin (S24) and chymotrypsin (S25), and 3) the largely unfolded bungarotoxin (S30). Interestingly, both lysozyme and papain CD spectra (Whitmore et al., 2011; Olamoyesan et al., 2021) have β_II_ characteristics suggesting this is a key to the problems with the first two types.

Overall, the solid state SOMSpec LOOV analysis can be described as being indicative of the secondary structure of the test protein as the errors are quite high. The increased accuracy when high helix, high sheet, and β_II_ proteins are removed flags a warning for the quality of the fitting for these classes of proteins. It should be noted that some of the reduced accuracy of the fits with the solid-state rather than film or solution proteins will be the result of the smaller reference set used. However, the space coverage of this reference set is fairly good so we attribute most of the increased error to the broader peaks.

### Gaussian Band-Fitting to Determine Secondary Structure

**FIGURE 4:**
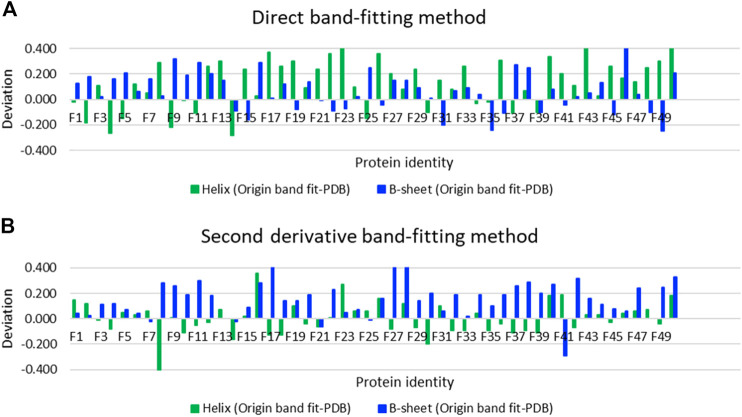
Deviations of secondary structure prediction from PDB structures for helix and *β*-sheet for the Amide I band of the 50-protein film reference set presented in order of decreasing helix content from left to right for **(A)** direct Gaussian band-fitting and **(B)** the second derivative fitting approach reported in reference (Yang et al., 2015). See Supplementary Material for protein identities. Deviations of the Other category deviations are minus the sum of helix and *β*-sheet deviations.

Using SOMSpec takes more effort than a simple band-fitting approach so we assessed whether the extra effort was worth it by making estimates of the secondary structures of the 50-protein thin-film reference set using both a direct Gaussian fitting and second derivative spectra fitting implemented in OriginPro. The differences between the predicted fractions of α-helix and β-sheet and the Protein data bank (PDB) (Berman et al., 2000) DSSP values (referred to as deviations) are plotted in Figure 4A for direct band-fitting and in Figure 4B for the Yang method *via* the second derivative spectra. The proteins have been plotted in order of helical content decreasing from left to right. The results of the direct fitting were average helix and sheet errors of 19 and 13%, respectively, whereas fitting *via* second derivatives gave average errors of 10 and 16%. We could see no patterns or common signatures of poor secondary structure estimates to help guide answering either question (i) or (ii) above.

## Conclusion

The main goal of this work was to establish a robust and reproducible approach, whose limitations are clear, to extract secondary structure information from Amide I protein IR spectra. In summary, we implemented our reference-set based self-organising map approach, SOMSpec, with a 50-protein thin-film reference set in both LOOV and on 68 other test proteins. We showed that the thin-film ATR spectra could be used as a reference set for transmission spectra aqueous proteins. The average SOMSpec prediction errors were 7% for both helix and sheet content for aqueous protein samples. High helix (>40–50%) estimates are of variable quality due e.g., to the high similarity of cytochrome-*c*’s spectrum (41% helix) and hemoglobin’s (77% helix). If high helix proteins are removed from the average, then the errors reduce to 5–6%. Due to the cause of the helix errors, adding more proteins to the reference set will not resolve it.

Problematic results were able to be identified by inspection of the SOMSpec outputs. In particular, shifts of wavelength maxima and loss of spectral intensity at high wavenumbers or low wavenumbers indicate, respectively, low helix and low sheet content in the prediction. We also found that proteins such as lysozyme and papain which have β_II_ characteristics in their CD spectra (Whitmore et al., 2011; Olamoyesan et al., 2021) have helix-like IR spectra. Finally, proteins with prosthetic groups which absorb in the Amide I region such as flavins and hemes may also cause errors in secondary structure predictions. Despite these caveats, a key advantage of the SOMSpec approach is that the fitting process is entirely reproducible so it can be used for batch-to-batch comparisons. The attraction of the 50-protein thin-film reference set is that the spectra mirror the shape of transmission spectra as illustrated in Figure 3C but are easier to collect and perform baseline correction than aqueous transmission spectra so the reference set itself is more reliable.

We also estimated the secondary structures of the 50-protein thin-film reference set using two band-fitting approaches and found that the errors can be significant and variable. This work and previous work by (Oberg et al., 2004) on applications of SELCON to IR data suggests that the key advantage of SOMSpec is that it is based on using a reference-set to provide secondary structure information. Thus, it (or e.g., SELCON3) is dependent on the quality of the reference set. SOMSpec has the additional advantage that it enables the user to interrogate the input and output regarding the quality of the fit.

A SOMSpec LOOV analysis of solid-state spectra suggests that there is enough information in solid-state spectra for useful secondary structure fitting, but that the 30-protein reference set is too small.

In accord with the results of Wi et al. (1998), we found that FSD does not improve structure fitting with the reference-set based SOM approach, though the spectral NRMSDs improved in a misleading manner. This is in accordance with FSD not actually increasing the information content of any spectrum.

Finally, ATR data collection is extremely attractive for aqueous protein samples as it is much simpler to mount the sample and simpler to perform the baseline correction. Although we have previously shown it is relatively straightforward to convert ATR spectra to transmission as summarised in the Supplementary Material (Rodger et al., 2020), many users find it attractive to be able to ignore the differences. Our conclusion for ATR data is that if the protein of interest either has high *β*-sheet content or an extra 2% average helix error is acceptable, then normalised ATR data can be used directly with a transmission (or ATR thin film as in this work) reference set, and conversely. As quality protein ATR data collection is much easier to achieve than with transmission, this innovation addresses some of the challenges of protein structure fitting from IR data.

## Data Availability

The original contributions presented in the study are included in the article/Supplementary Materials, further inquiries can be directed to the corresponding author.

## Supplementary Material

Supplementary Data Sheet S1 Supplementary material text (including details of SOMSpec, protein identities and band fitting methods).

Supplementary Data Sheet S2 Film 50 set input and LOOV output inc band fitting with plots at side.

Supplementary Data Sheet S3 30-solid state Input data and output predictions plots at side.

Supplementary Data Sheet S4 Aq trans & ATR 21&47 output tested against 50-film map with test plot.
