# Supplementary material for: SOMSpec as a General Purpose Validated Self-Organising Map Tool for Rapid Protein Secondary Structure Prediction From Infrared Absorbance Data
Source: Front Chem. 2022 Jan 27;9:784625. doi: 10.3389/fchem.2021.784625 (PMC8830495; doi:10.3389/fchem.2021.784625)
Supplement: Supplementary file 3 [file DataSheet1.PDF]

frontiers

Supplementary Material

1 Data Availability Statement

- This supplementary information expands on the SOMSpec methodology, ATR to and from transmission conversion, Fourier self-deconvolution, the identity of the proteins in the 30-protein solid state, 50 protein film, and test reference sets, and gives the protocols used for the direct band-fitting method and the derivative approach adopted based on reference (Yang et al., 2015).

. The Output from the calculations are given in the spreadsheets. All datasets generated and analysed for this study can be found at https://figshare.mq.edu.au/account/projects/122597/articles/16641538. Files are available to provide the data that has been plotted in the main paper and sufficient examples to enable SOMSpec to be implemented with other data sets. The Repository files are organised as follows:

- <SOMSpec code> contains the files required to run SOMSpec
- <30-solid state reference set> provides everything related to this reference set
- <50-film reference set> includes the LOOV output summary, trained map, the full reference set, training input file, a trained map, the direct and derivative band-fitting output summary.
- <Aqueous transmission and ATR testing input and output> contains a spreadsheet (with the 21 aqueous transmission and ATR spectra and the summary of SOMSpec output), test input files, and SOMSpec output.
- <Bandfitting1> provides the output files and an output summary for the bandfitting methods used.

2 Self-organising map for spectra fitting: SOMSpec

A self-organizing map (SOM) is a neural network architecture created by Kohonen that produces a 2D representation from a higher dimensional input space and thus helps the visualisation and identification of structures in data (Kohonen, 1982). We previously created a SOM approach to CD structure fitting (Hall et al., 2013;Hall et al., 2014a;Hall et al., 2014b) which we have now generalised to be used for IR and called SOMSpec (Corujo et al., 2018). In our case, the 2D representation consists of a rectangular map of nodes each of which has a spectrum assigned to it and a second map which has the corresponding secondary structures (SS) assigned to the same node. Our input for training the map is columns of data (formatted as comma separated *txt* files) with each column being a vector of spectral data appended with its secondary structure annotations. Once a map has been trained and assigned, it can be used repeatedly. Test files are the same as training files but without the structure assignments. The MATLAB code for SOMSpec as currently written needs the test spectra and reference spectra (vectors of spectral data) to have the same upper and lower wavenumbers (or wavelengths in the case of CD) but can have any step-size.

The trained map is produced by identification of the best matching unit (BMUs) for each reference vector followed by changing it and all other nodes within the current neighbourhood according to

$$W(t+1) = W(t) + \theta(t)L(t)[I(t) - W(t)] \quad (1)$$

where, t is the current iteration and $(t+1)$ the one that follows, $\theta$is the radial bias function

$$\theta(t) = exp\frac{-D(t)^2}{2R(t)^2} \quad (2)$$

where D is the radial distance from the BMU and R the distance at which the value of the function is 1/√e and thus the parameter that defines how rapidly the influence of the node drops across the map. L is the learning rate and means the extent of learning for each iteration (we used 0.1). Both the radius and learning rates, Z, are set to decay exponentially over iterations

$$Z(t+1) = Z(t)exp\frac{-t}{2R(t)^2c} \quad (3)$$

where the time constant c determines the rate at which Z drops each iteration. It is calculated from the total number of iterations (*Iter*) and initial radius (R_o) by

$$c = Iter/lnR_o \quad (4)$$

The process is repeated for the selected number of iterations.

During the structure assignment, the input reference vectors are sequentially sampled against the map and a BMU assigned based on a minimised Normalized Residual Mean Square Difference

$$NRMSD = \frac{\left(\sqrt{\frac{\Sigma(Y-X)^2}{N}}\right)}{(M-m)} \quad (5)$$

where N is the number of data points, M the maximum value, and m the minimum value. The denominator for calculating spectral NRMSDs for normalised protein IR spectra can be ignored. The protein and its SS content are assigned to that node and the surrounding nodes have SS content assigned based on their distance from the known proteins.

Finally, the test spectrum is put through the algorithm in order to determine its SS. A predefined number of BMUs (usually 5 or 3) are ranked by similarity and the property contribution of each calculated using a distance dependent weighting. An output of the trained map is generated for visualization with the BMUs (in red), the test experimental spectrum, the predicted spectrum from the weighed sum of BMU spectra and the corresponding spectral NRMSD value.

In this work we used a 20×20 or 40×40 map size (previous work suggested a map size somewhat smaller than the reference set size is optimal), 20,000 iterations for training the LOOV maps and 50,000 training maps for testing unknowns, 5 or 6 secondary structures per protein (depending on the annotation of the reference data and in any case reduced to 3 for discussion), usually 3 BMUs per prediction, and wavenumber range 1600 – 1800 cm^{-1}.

The SOMSpec code and examples of formatted input training and testing files is provided in the ESI. It requires MATLAB to run. To view files, it may be convenient to replace <*.txt*> by <*.csv*>. It is important to ensure there are no stray characters in the files as this results in errors.

To proceed, create *Train* and *Test* files in .txt format. Usually, they are most easily generated *via* .csv format in Excel, then simply renamed .txt. Then launch <*SOMSpecGUI.mlapp*>. Wait for the second MATLAB window to open. To use a trained map available in the ESI, make sure the <*Train*> tab has the correct Map size, # iterations 20000, # structures, # BMUs, Wavelength range and Units. If the right-hand side of the <*Train*> tab does not reflect the left-hand side entries change an entry and change it back. Then on the <*Predict*> tab, select the pretrained SOM of choice. Select the input spectral file of interest, uncheck and re-check <*Disable scaling of spectra*>, and click <*Run prediction*>. If you get an error message, check the <*.txt*> file formats.

3 Transforming transmission spectra into ATR spectra

We inverted the methodology developed in reference (Rodger et al., 2020) to convert transmission spectra into what would be collected on the same sample with a 45° incidence ZnSe ATR crystal.

$$A_{ATR} = log_{10}\left(1 + ln10\epsilon C d_p f\right) \tag{6}$$

where A_{ATR} denotes the ATR absorbance, $d_p f$ is the penetration depth times the light intensity factor and $\varepsilon C = A/\ell$ is the extinction coefficent times concentration which for a water plus protein solution is the sum of that for the two components. As the protein absorbance is much smaller than that of water in our experiments, we used $d_p f$ for water and truncated the expansion of equation (1) at second order in water absorbance and first order in protein. We can therefore approximate the ATR protein absorbance as

$$A_{ATRprotein} = (\epsilon C)_{protein}\left(d_p f\right)\left(1 - \left(ln10 d_p f\right)(\epsilon C)_{water}\right) \tag{7}$$

which conveniently does not require us to know the protein concentration or extinction coefficent if we ultimately wish to normalise the ATR spectrum for structure fitting. A spreadsheet is provided (<ESI 19 of Transmission to ATR conversion & 2 of ATR to trans aq IR>) that uses equation (2) and the wavelength dependence of $d_p f$ and $(\epsilon C)_{water}$ from reference (Rodger et al., 2020) to calculate the ATR spectra for a single bounce 45° incidence ZnSe crystal for 19 test proteins. It also includes conversion of 2 high β-content ATR spectra to transmission.

4 Fourier self-deconvolution

The effect of Fourier self-deconvolution performed in Origin Pro with different smoothing factors and γ parameters is illustrated in Figure SM1.

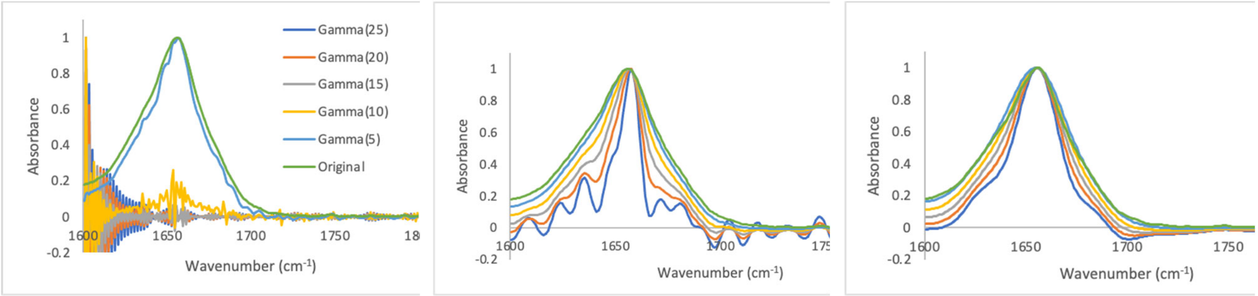

Figure SM1. Original and FSD spectra of bovine serum albumin with FSD smoothing factors 0, 0.25, and 0.50 (left to right) and γ varied between 25 and 5.

5 Proteins used in this work

Table SM1. The sets of protein data used in this work with α-helix or total helix (where α and 3_{10} were used in the annotation) and β-sheet content indicated. The BioTools reference set is available from BioTools (Jupiter, Florida). Expt. Max. is the wavenumber maximum of the experimental spectrum. For the 21 worse fits (above 12% deviation from crystal for either helix or β-sheet), the final column gives the reason, where (i)–(vii) refer to the list in section 3.1 of the main paper.

**50 Dried thin film proteins (reference (Goormaghtigh et al., 2006))**		**total helix**	**β-sheet**	**Expt. Max.**	**Poor fit reason**
F1	Myoglobin	0.74	0	1652 cm^{-1}	
F2	Colicin A-C-terminal domain	0.76	0	1654 cm^{-1}	
F3	Apo-Ferritin	0.73	0	1651 cm^{-1}	
F4	Haemoglobin	0.77	0	1653 cm^{-1}	(iii)
F5	Apolipoprotein E3	0.65	0	1653 cm^{-1}	
F6	Troponin	0.64	0.04	1651 cm^{-1}	+1 (iv)
F7	Citrate synthetase	0.61	0.02	1655 cm^{-1}	–0.5 cm^{-1}
F8	Parvalbumin	0.58	0.03	1952 cm^{-1}	
F9	Glutathione-S-transferase	0.5	0.09	1651 cm^{-1}	
F10	Fe superoxide dismutase	0.52	0.11	1653 cm^{-1}	(ii)
F11	Phospholipase A2	0.49	0.07	1653 cm^{-1}	–5 cm^{-1} (iv)
F12	Cytochrome c	0.41	0	1654 cm^{-1}	(ii)
F13	Insulin	0.47	0	1653 cm^{-1}	
F14	Triose Phosphate Isomerase	0.45	0.16	1643 cm^{-1}	
F15	Dihydropteridine reductase	0.40	0.23	1655 cm^{-1}	(v)
F16	Peroxidase	0.42	0.05	1651 cm^{-1}	
F17	Phosphoglycerate kinase	0.34	0.11	1639 cm^{-1}	High NRMSD
F18	Lipoxidase	0.39	0.13	1651 cm^{-1}	
F19	Transpeptidase	0.34	0.17	1653 cm^{-1}	
F20	Penicillin Amidohydrolase	0.35	0.19	1647 cm^{-1}	–3 cm^{-1} (vii)

F21	Lysozyme	0.42	0.06	1652 cm^{-1}	
F22	Subtilisin Carlsberg	0.31	0.17	1648 cm^{-1}	
F23	alpha-Lactalbumin	0.43	0.07	1652 cm^{-1}	
F24	Subtilisin BPN9	0.29	0.17	1649 cm^{-1}	
F25	Ovalbumin	0.31	0.31	1639 cm^{-1}	
F26	Glucose Oxidase F2	0.34	0.20	1651 cm^{-1}	(ii)
F27	Alcohol Dehydrogenase	0.29	0.24	1639 cm^{-1}	(iv)
F28	Papain	0.26	0.18	1649 cm^{-1}	
F29	Ricin	0.23	0.25	1648 cm^{-1}	
F30	Ribonuclease A	0.21	0.33	1641 cm^{-1}	+4 cm^{-1}
F31	Monellin	0.17	0.52	1636 cm^{-1}	
F32	Ubiquitin	0.25	0.32	1642 cm^{-1}	
F33	Pancreatic Trypsin Inhibitor	0.21	0.24	1647 cm^{-1}	+6 cm^{-1}
F34	Pepsin	0.15	0.43	1640 cm^{-1}	
F35	Rennin	0.15	0.46	1640 cm^{-1}	
F36	Pepsinogen	0.21	0.39	1640 cm^{-1}	
F37	Thaumatin	0.11	0.36	1639 cm^{-1}	
F38	Carbonic Anhydrase	0.17	0.29	1637 cm^{-1}	(v)
F39	Chymotrypsinogen A	0.13	0.32	1637 cm^{-1}	+3 cm^{-1} (v)
F40	Trypsinogen	0.1	0.31	1638 cm^{-1}	+5 cm^{-1} (v)
F41	Xylanase	0.05	0.62	1632 cm^{-1}	+3 cm^{-1} (v)
F42	Immunoglobulin G	0.08	0.47	1639 cm^{-1}	–3 cm^{-1} (vi)
F43	Superoxide dismutase	0.07	0.39	1637 cm^{-1}	
F44	Lentil Lectin	0.03	0.48	1634 cm^{-1}	
F45	Alpha Toxin	0.04	0.56	1634 cm^{-1}	
F46	Soy Trypsin Inhibitor	0	0.25	1641 cm^{-1}	–5 cm^{-1}
F47	Avidin	0.04	0.46	1633 cm^{-1}	
F48	Erabutoxin	0	0.44	1638 cm^{-1}	(v)
F49	Concanavalin A	0.04	0.46	1633 cm^{-1}	
F50	Metallothionein II	0	0	1652 cm^{-1}	(vii)
**21 aqueous test proteins: 19 Transmission + 2 ATR**		**α-helix**	**β-helix**		
T1	Haemoglobin	0.77	0.00		
T2	Bovine serum albumin	0.73	0.00		
T3	Bovine fibrinogen	0.53	0.10		
T4	Peroxidase	0.49	0.04		
T5	Aldolase	0.45	0.15		
T6	Alpha lactalbumin	0.44	0.11		
T7	Hexokinase	0.41	0.16		
T8	Lysozyme	0.41	0.11		
T9	Apotransferrin	0.34	0.17		
T10	Glyceraldehyde	0.31	0.27		
T11	Deoxyribonuclease	0.29	0.29		
T12	Papain	0.26	0.22		
T13	Ribonuclease	0.21	0.35		

T14	Trypsin inhibitor	0.20	0.24		
T15	Carbonic anhydrase	0.16	0.30		
T16	Beta Lactoglobulin	0.13	0.39		
T17	alpha Chymotrypsin	0.12	0.34		
ATR18	an IGG	0.07	0.47		
ATR19	Laminin G-like domain 3 from human perlecan	0.06	0.45		
T20	Concanavalin A	0.04	0.45		
T21	Trypsin	0.02	0.40		
**30 Solid state proteins**		**α-helix**	**β-sheet**		
S1	Myoglobin	0.79	0		
S2	Haemoglobin from bovine	0.76	0		
S3	Albumin bovine	0.74	0		
S4	Albumin human	0.70	0		
S5	Peroxidase from horseradish	0.49	0.02		
S6	Insulin human	0.47	0		
S7	Alpha lactalbumin	0.46	0.07		
S8	Deoxyribonuclease I from bovine pancreas	0.45	0.17		
S9	Phosphatase alkaline bovine	0.44	0.14		
S10	Hexokinase Saccharomyces	0.40	0.16		
S11	Cytochrome c bovine	0.40	0.11		
S12	Lysozyme chicken	0.40	0.06		
S13	Actin from bovine muscle	0.36	0.23		
S14	Glyceraldehyde-3-phosphate dehydrogenase rabbit	0.36	0.23		
S15	Transferrin apo human	0.34	0.16		
S16	Catalase bovine	0.32	0.16		
S17	D amino acid oxidase	0.31	0.28		
S18	Alpha amylase *bacillus licheniformis*	0.27	0.23		
S19	Papain papaya latex	0.25	0.16		
S20	Ribonuclease A bovine	0.21	0.33		
S21	Beta lactoglobulin bovine milk	0.17	0.41		
S22	Carbonic anhydrase isozyme II bovine	0.16	0.29		
S23	Gamma globulins	0.15	0.35		
S24	Trypsin bovine	0.10	0.31		
S25	Alpha chymotrypsin bovine	0.08	0.32		
S26	Superoxide dismutase bovine	0.06	0.39		
S27	Lectin from *Phaseolus Vulgaris*	0.05	0.41		
S28	Concanavalin A	0.04	0.		
S29	Alpha bungarotoxin	0.0	0.5		
S30	Trypsin inhibitor	0.0	0.36		

6 SOMSpec output for proteins F1–F50

F1

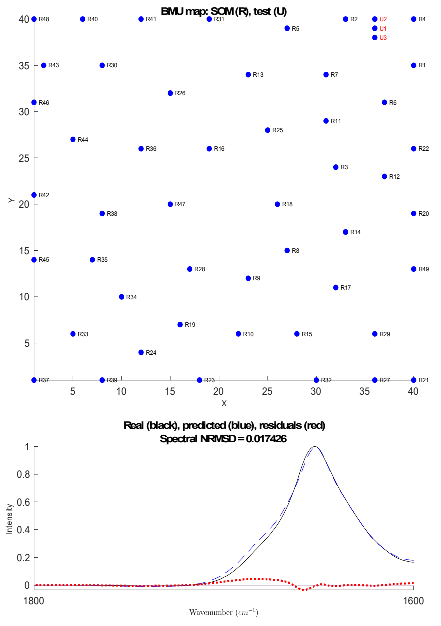
BMU map: SOM (R), test (U)
Real (black), predicted (blue), residuals (red)
Spectral NRMSD = 0.017426
Intensity
Wavenumber (cm^{-1})

F2

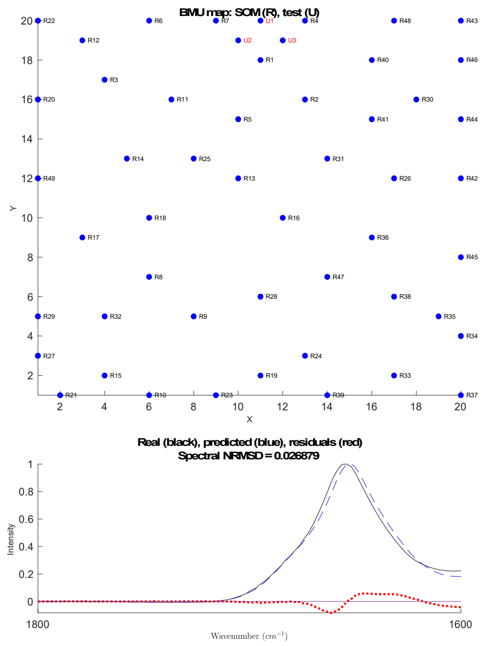
BMU map: SOM (R), test (U)
Real (black), predicted (blue), residuals (red)
Spectral NRMSD = 0.026879
Intensity
Wavenumber (cm^{-1})

F3

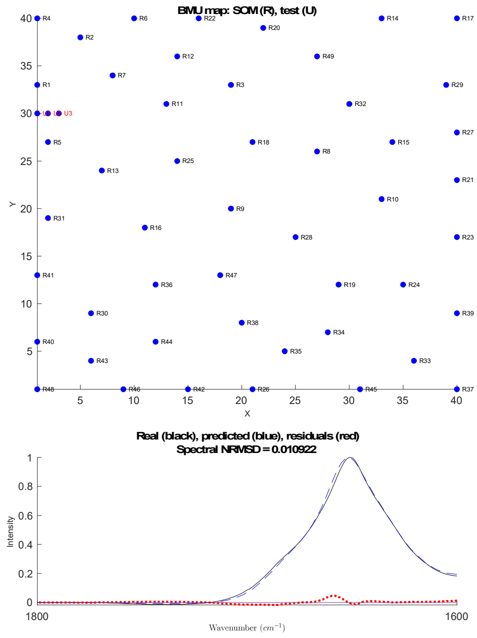
BMU map: SOM (R), test (U)
Real (black), predicted (blue), residuals (red)
Spectral NRMSD = 0.010922
Intensity
Wavenumber (cm^{-1})

F4

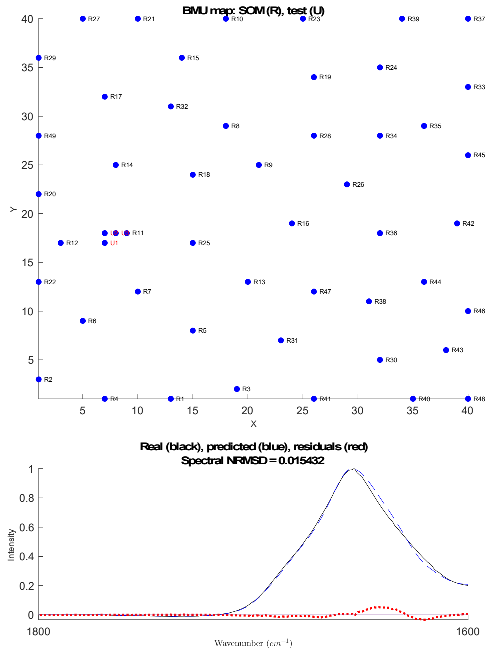
BMU map: SOM (R), test (U)
Real (black), predicted (blue), residuals (red)
Spectral NRMSD = 0.015432
Intensity
Wavenumber (cm^{-1})

F5

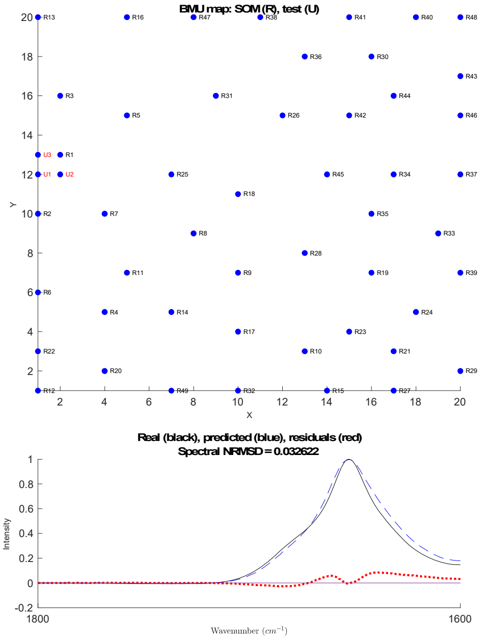
BMU map: SOM (R), test (U)
Real (black), predicted (blue), residuals (red)
Spectral NRMSD = 0.032622
Intensity
Wavenumber (cm^{-1})

F6

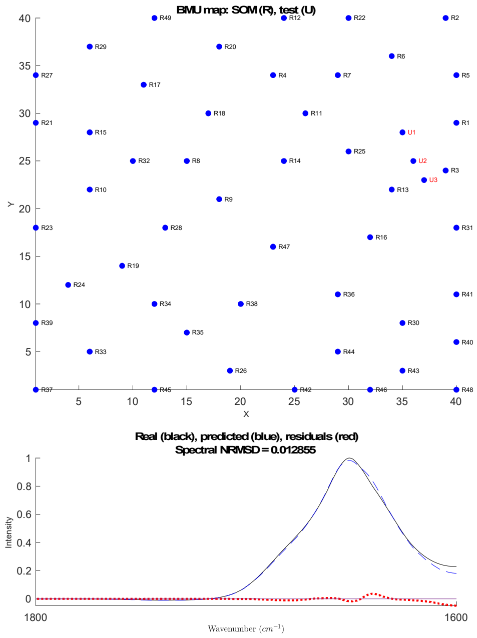
BMU map: SOM (R), test (U)
Real (black), predicted (blue), residuals (red)
Spectral NRMSD = 0.012855
Intensity
Wavenumber (cm^{-1})

F7

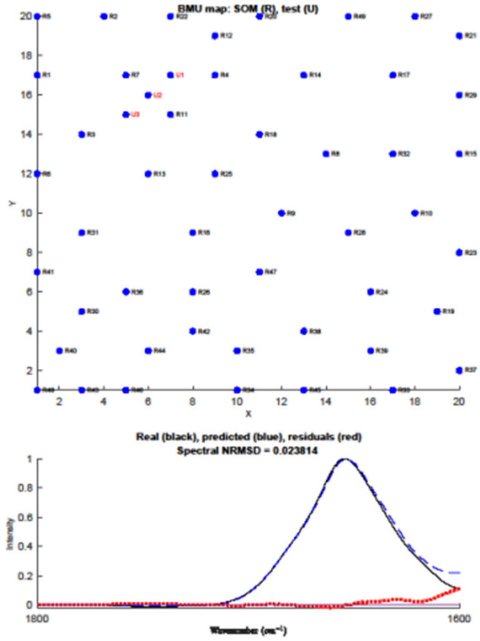
BMU map: SOM (R), test (U)
Real (black), predicted (blue), residuals (red)
Spectral NRMSD = 0.023814

F8

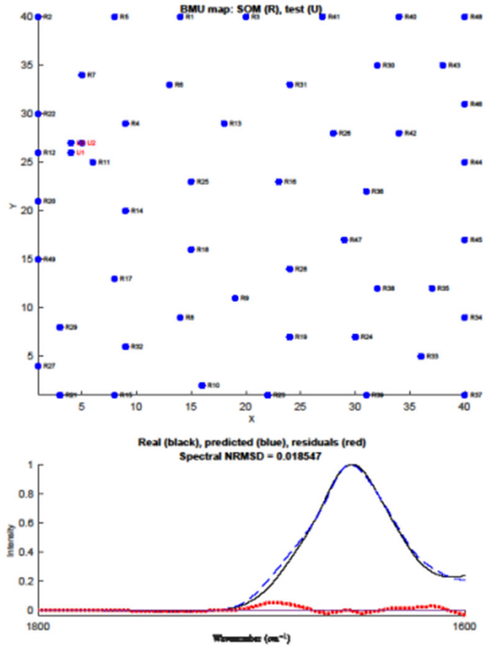
BMU map: SOM (R), test (U)
Real (black), predicted (blue), residuals (red)
Spectral NRMSD = 0.018547

F9

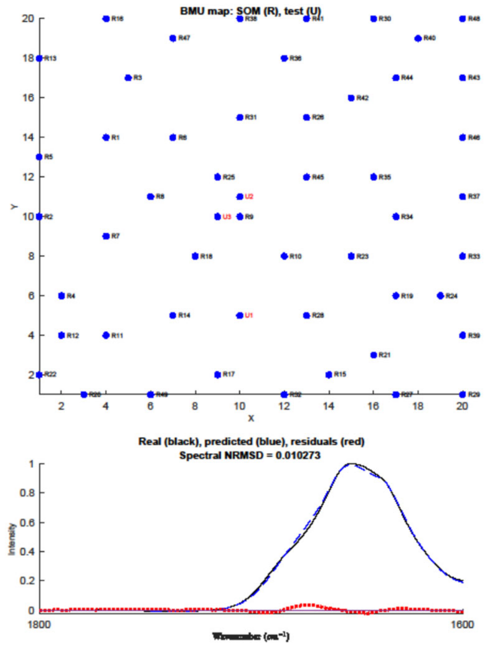

F10

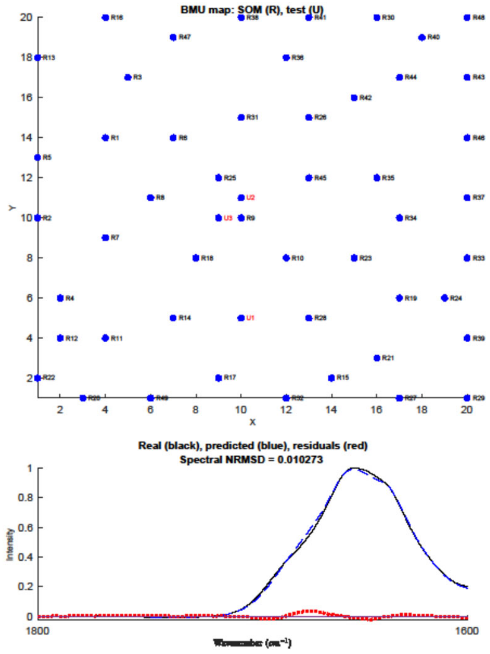

F11

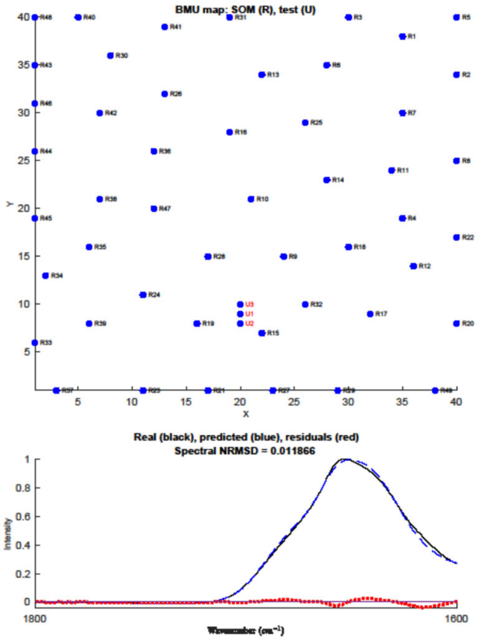

F12

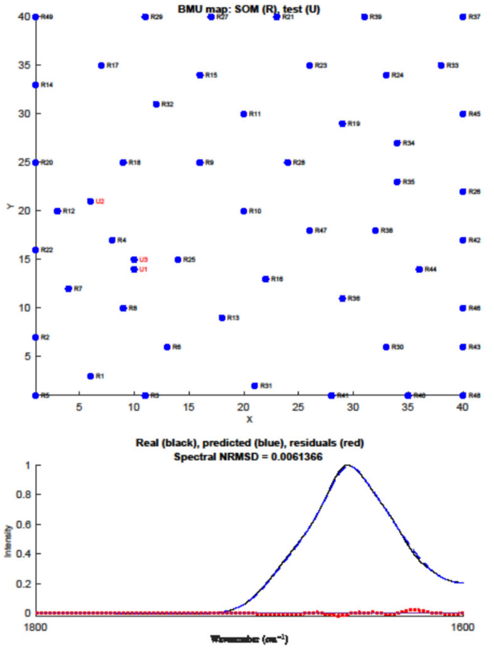

F13

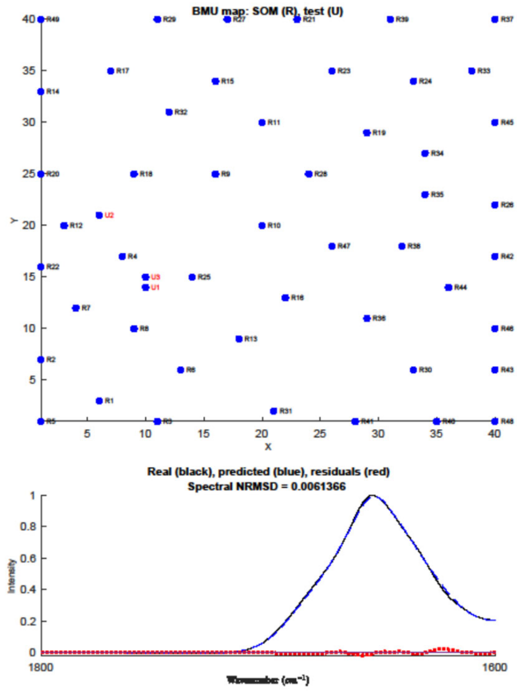

F14

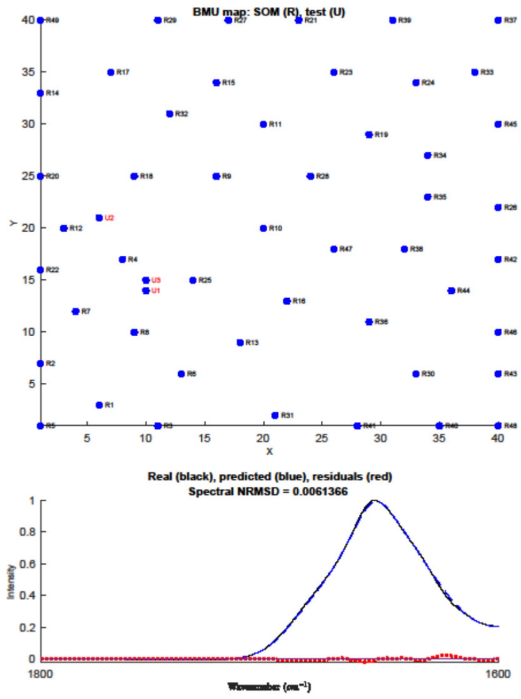

F15

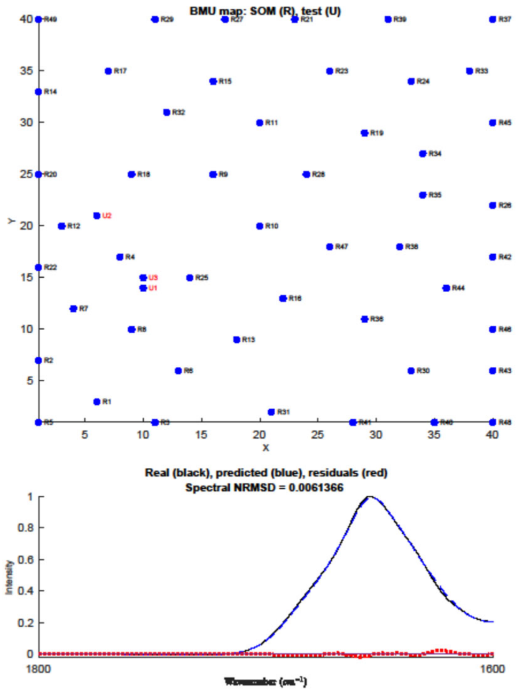

F16

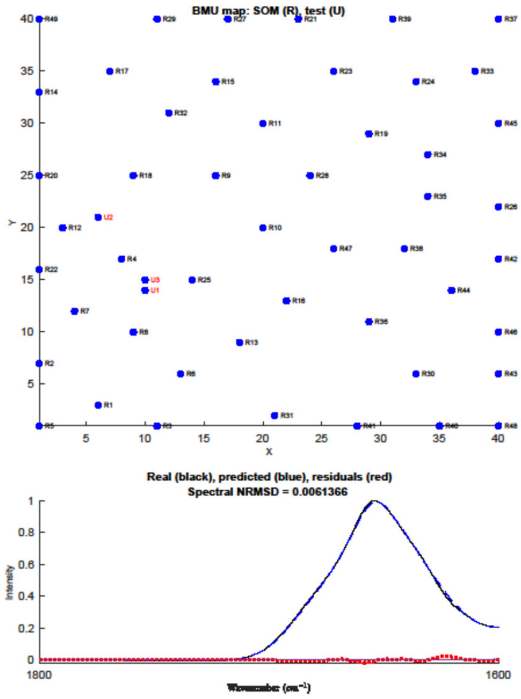

F17

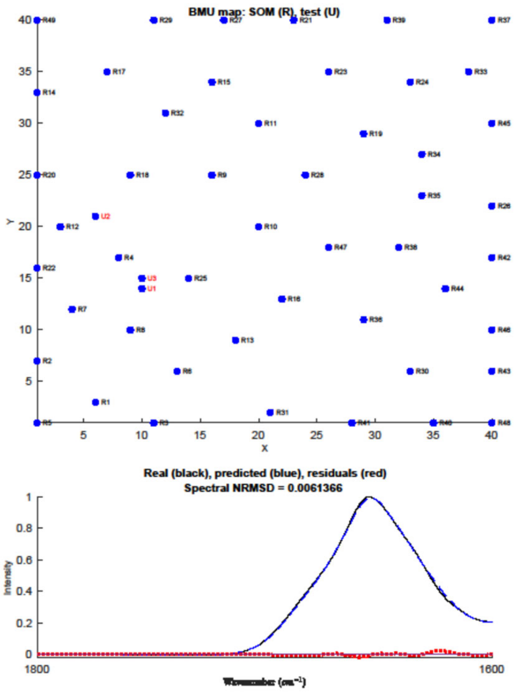

F18

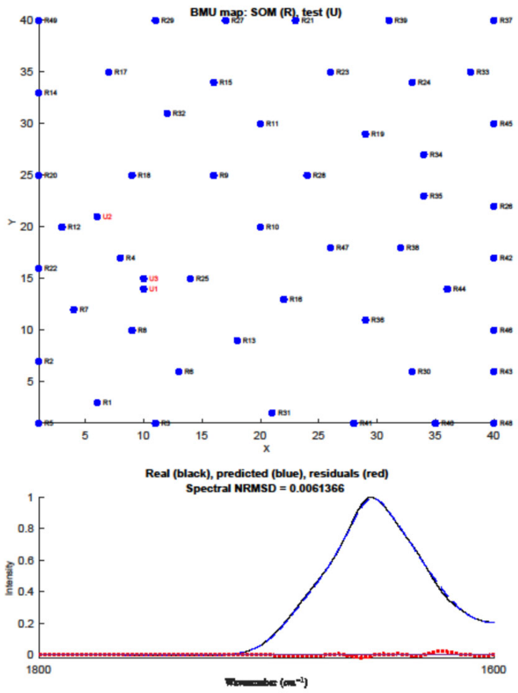

F19

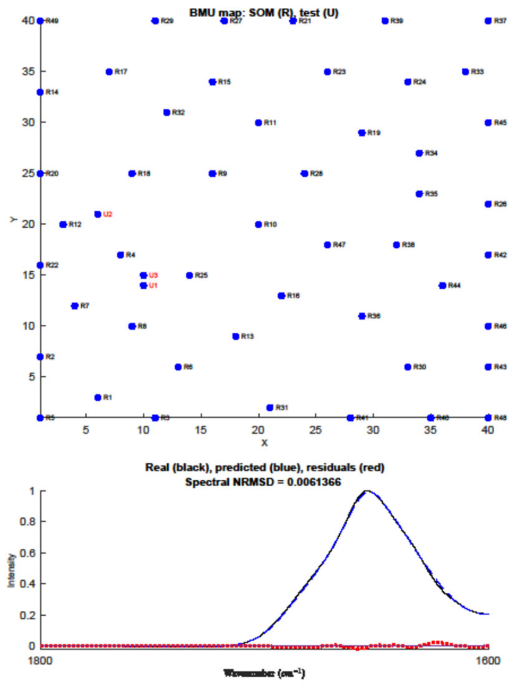

F20

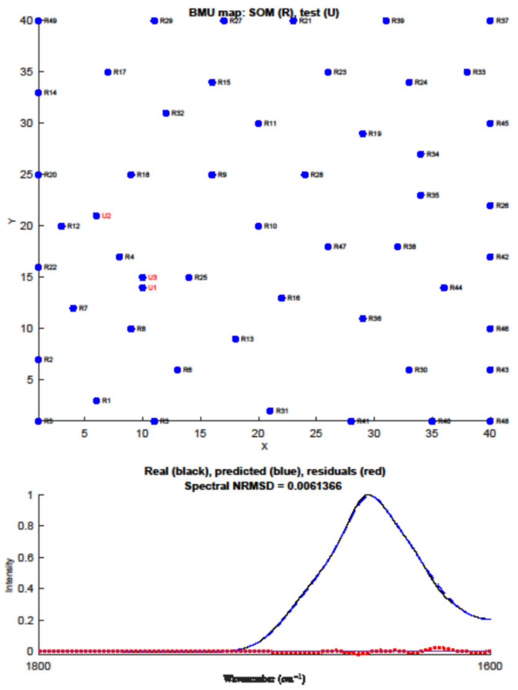

F21

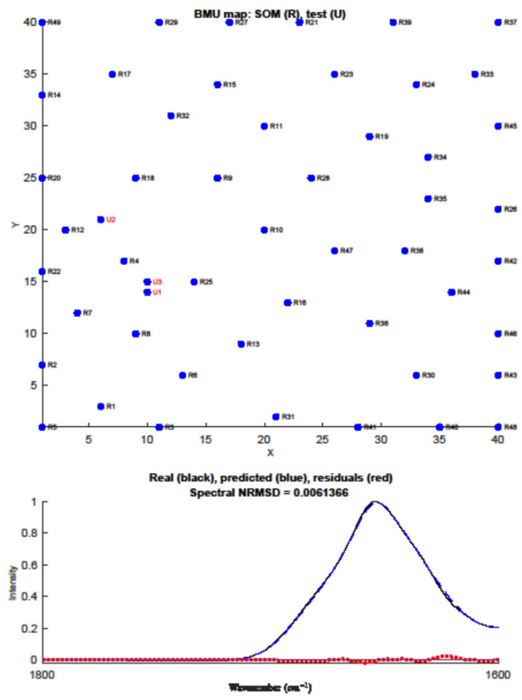

F22

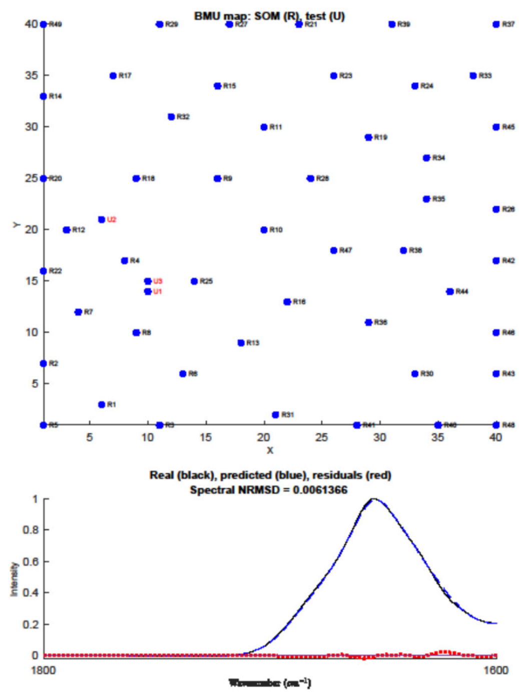

F23

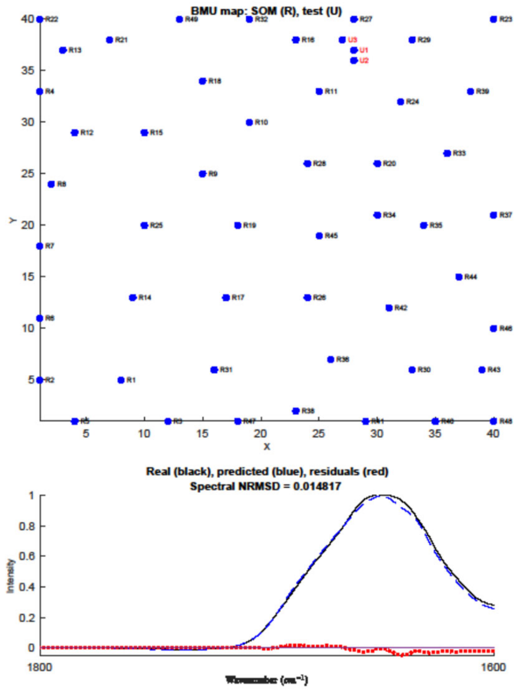

F24

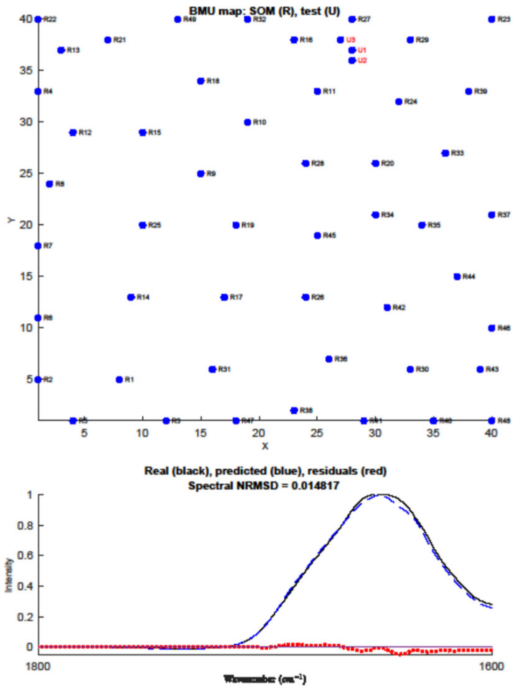

F25

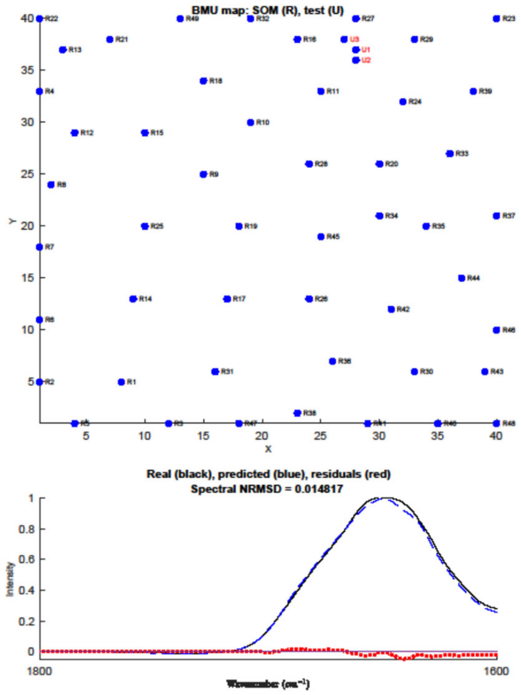

F26

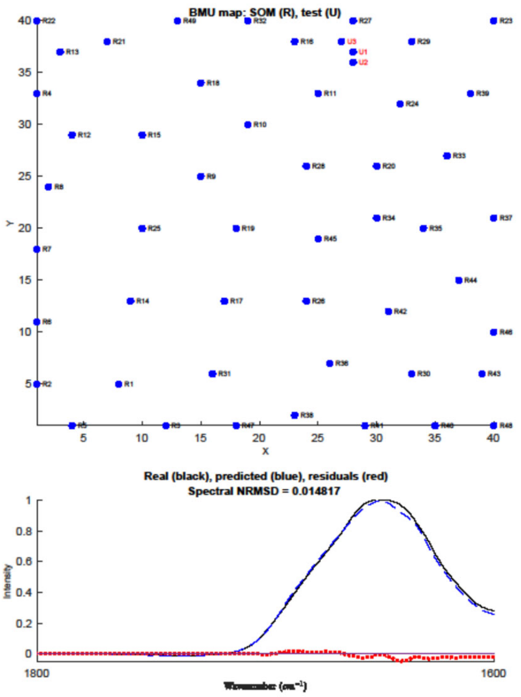

F27

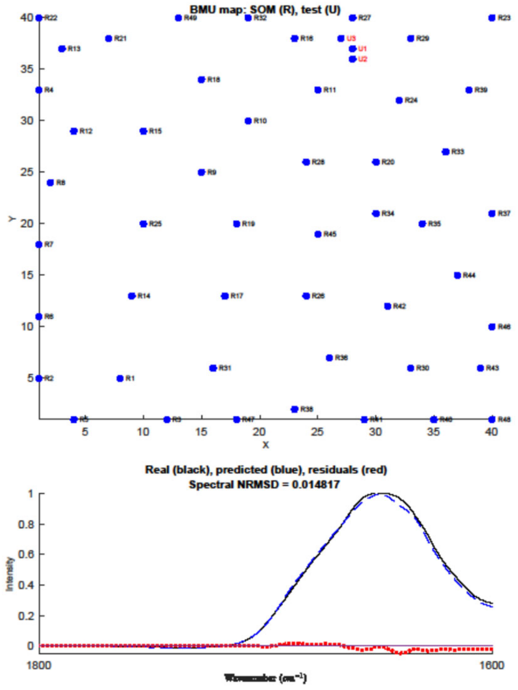

F28

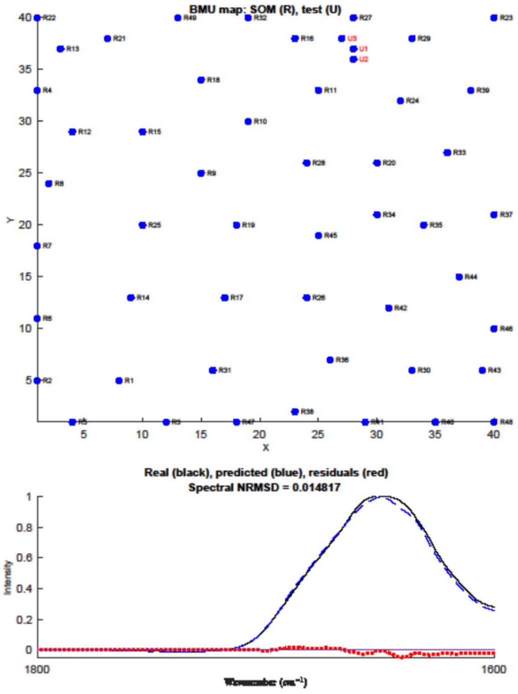

F29

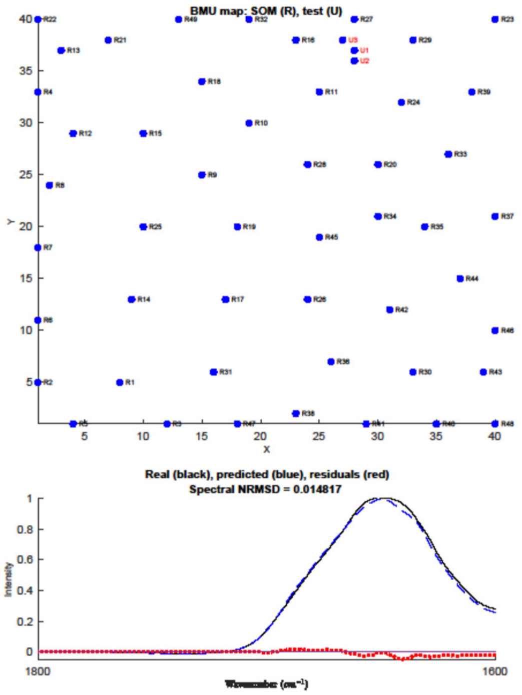

F30

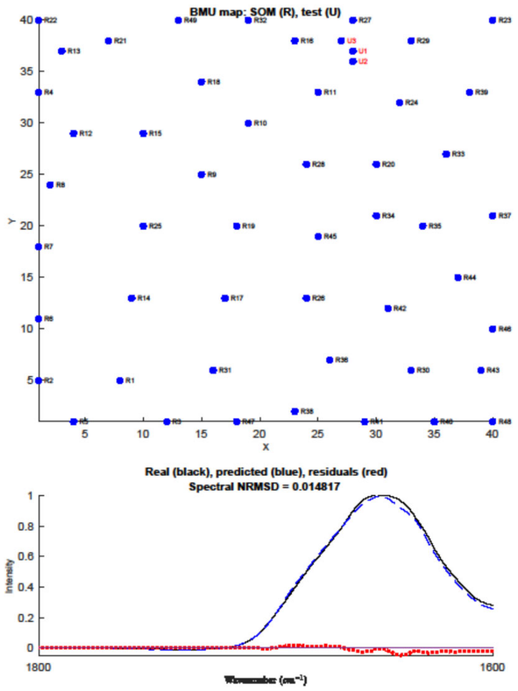

F31

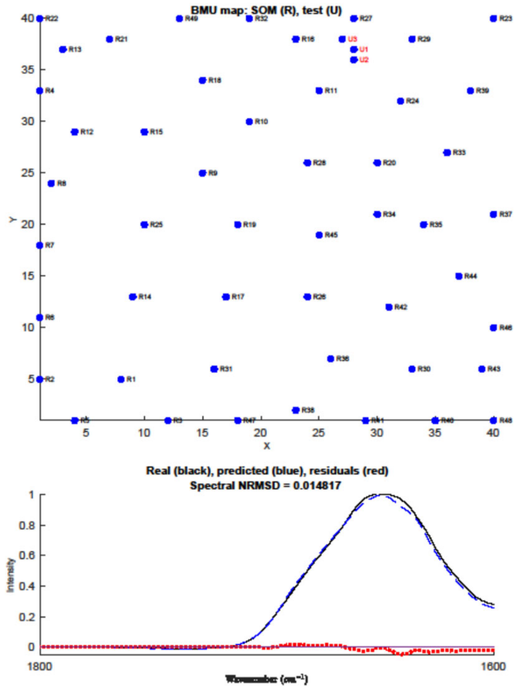

F32

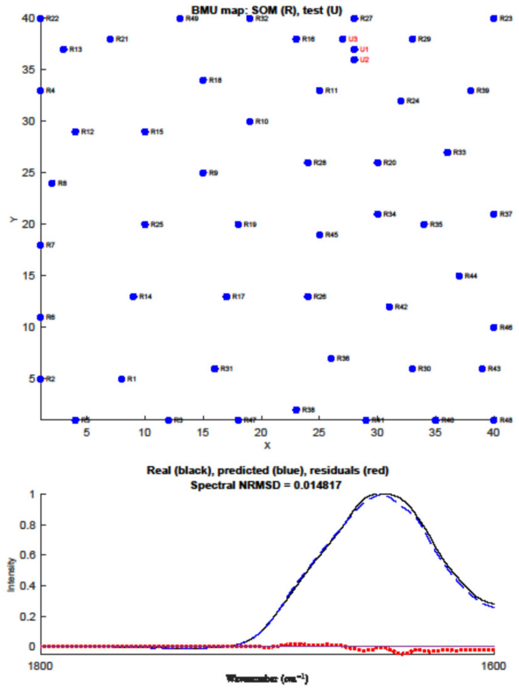

F33

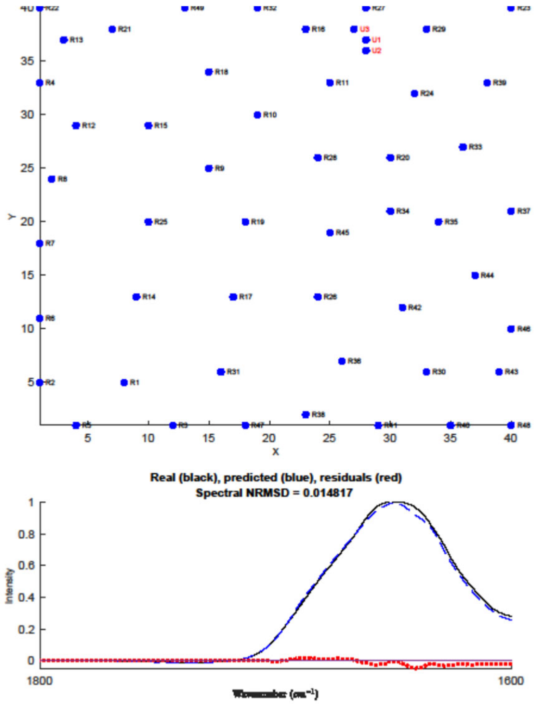

F34

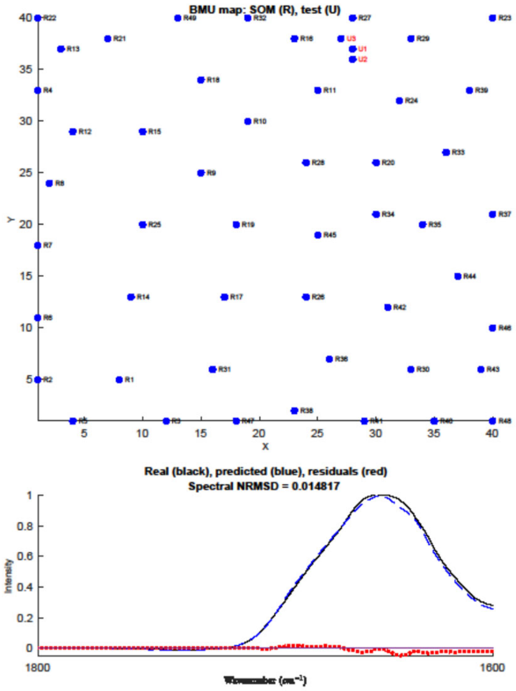

F35

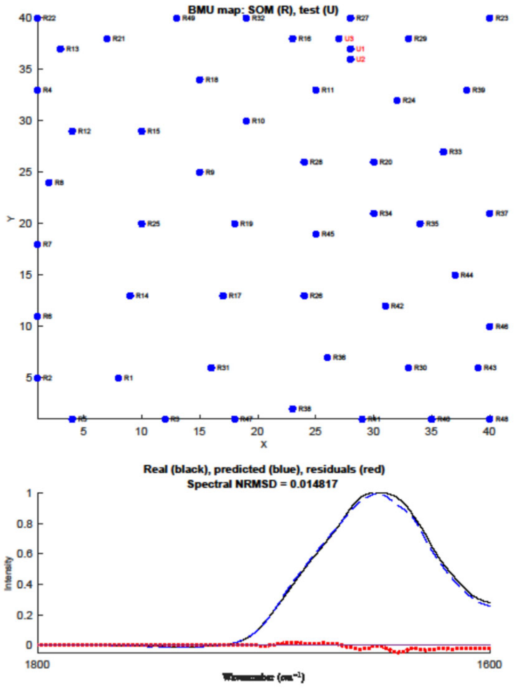

F36

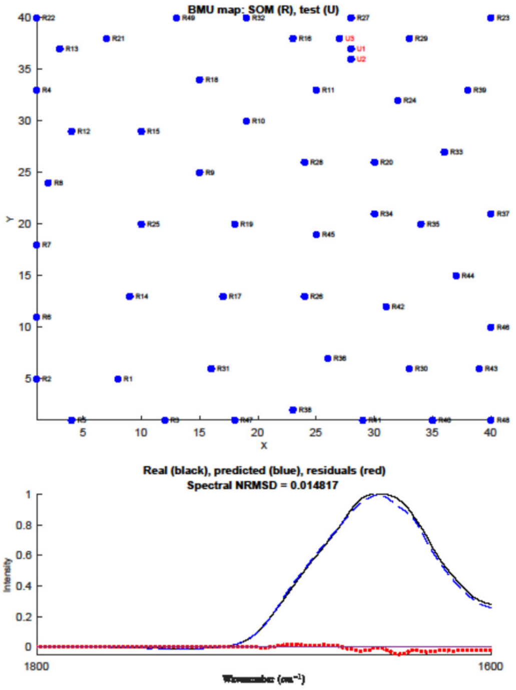

F37

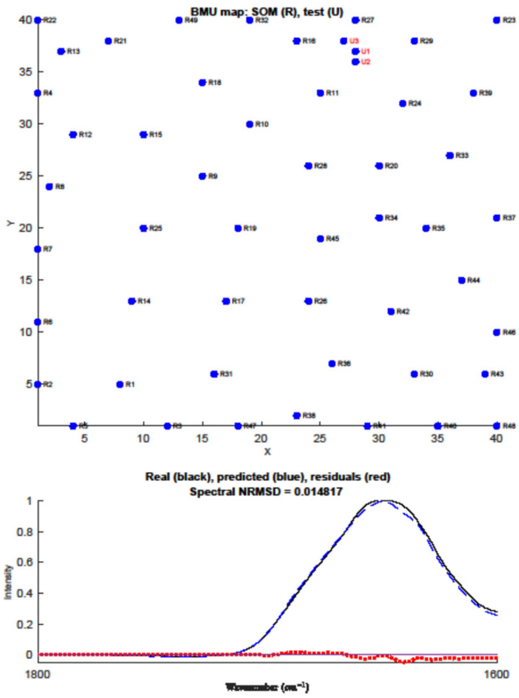

F38

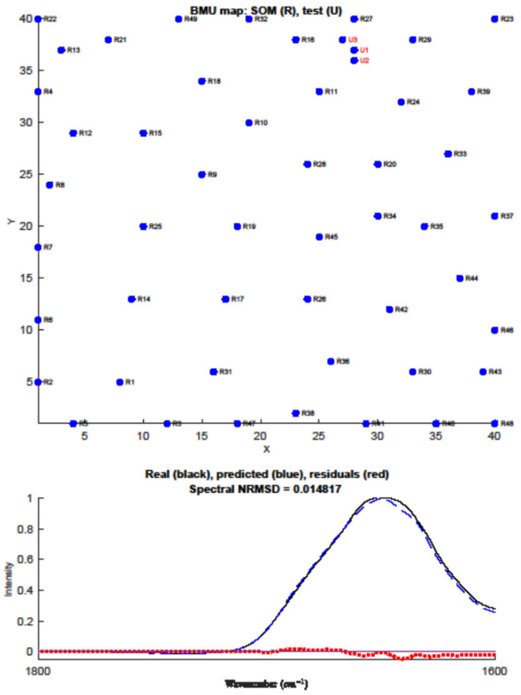

F39

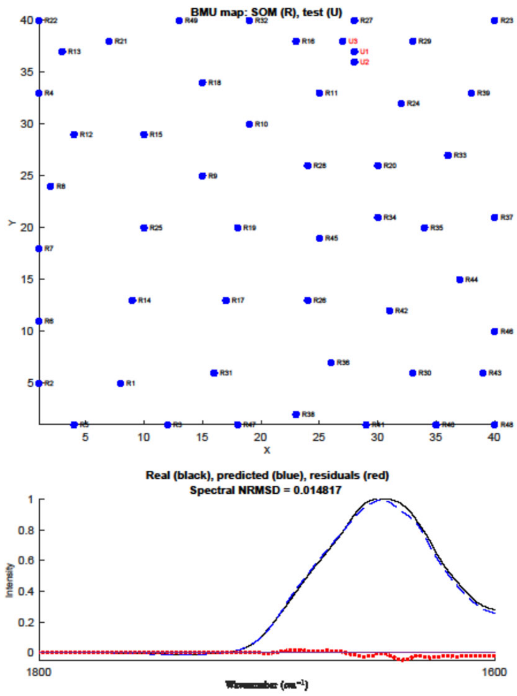

F40

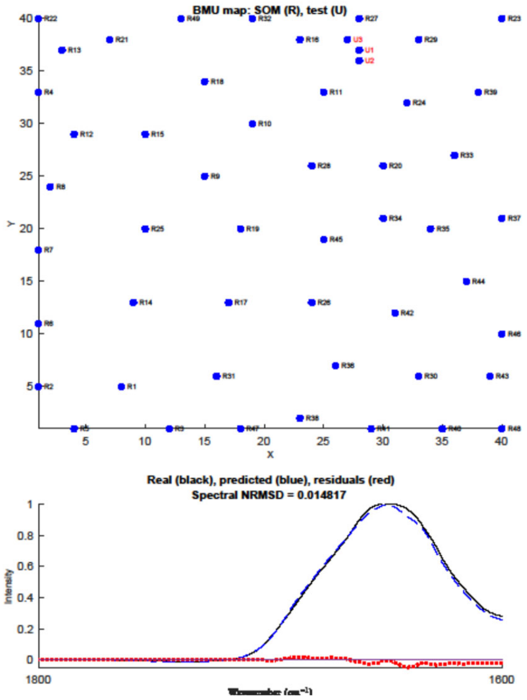

F41

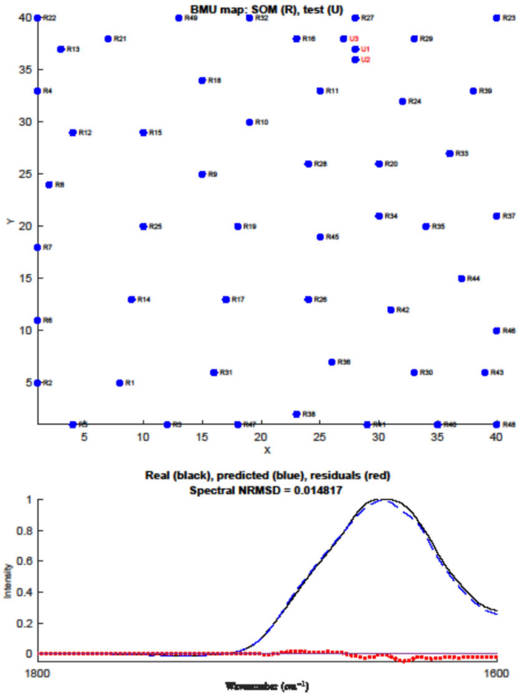

F42

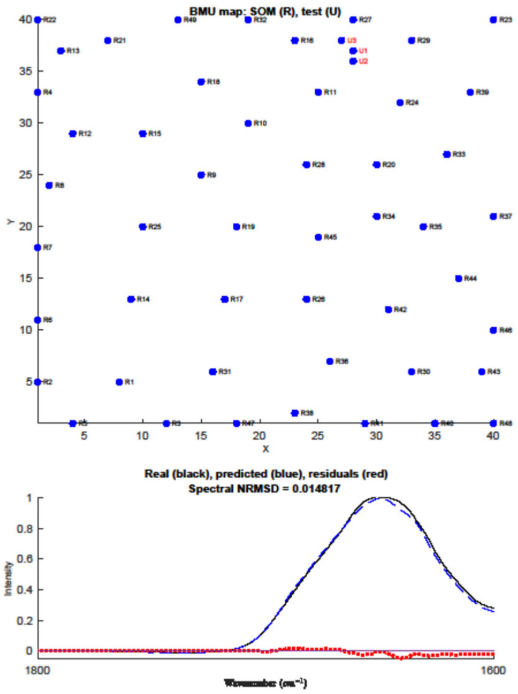

F43

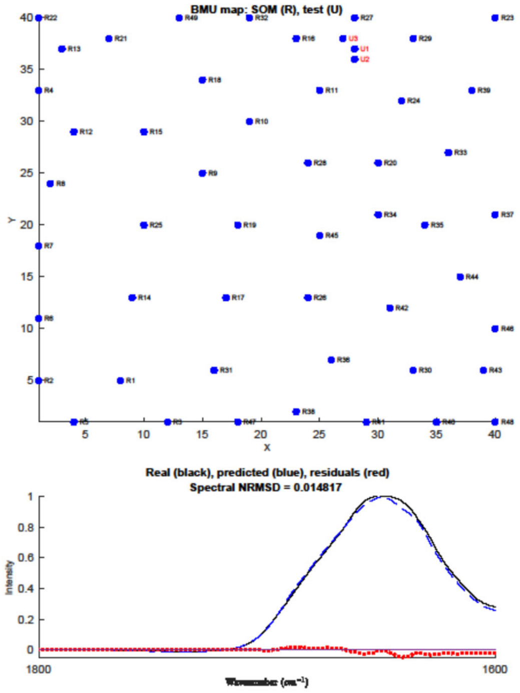

F44

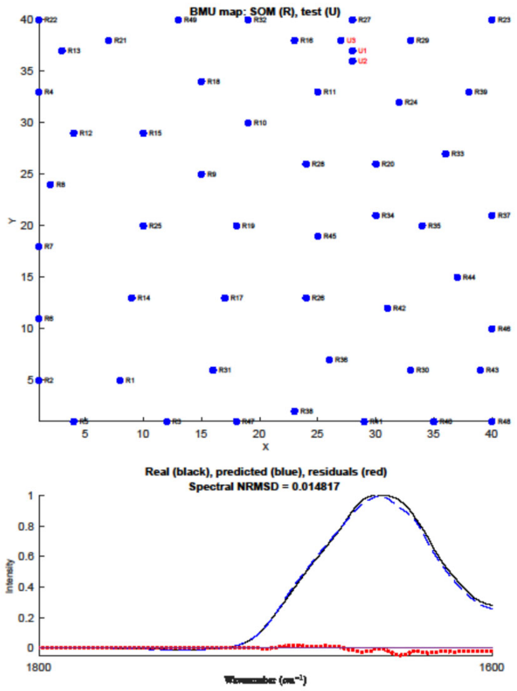

F45

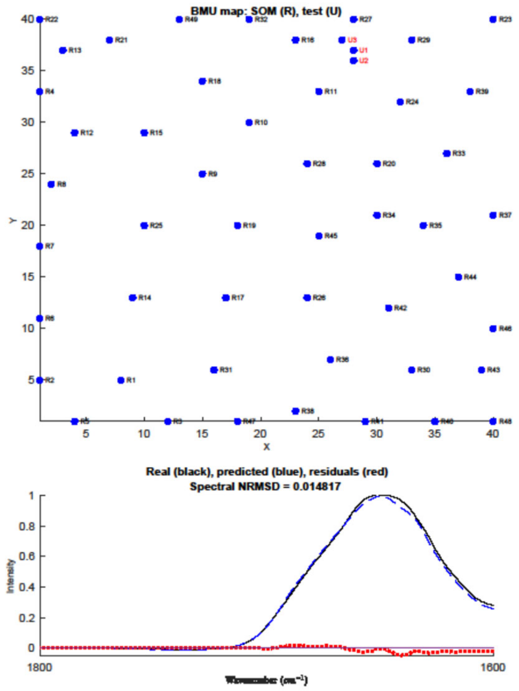

F46

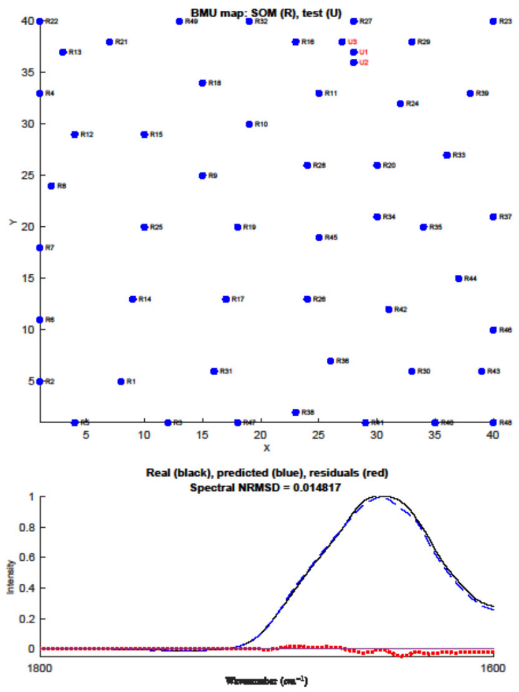

F47

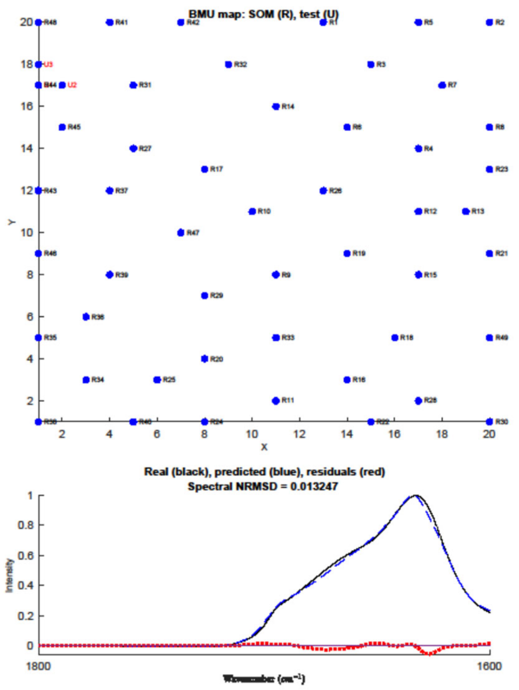

F48

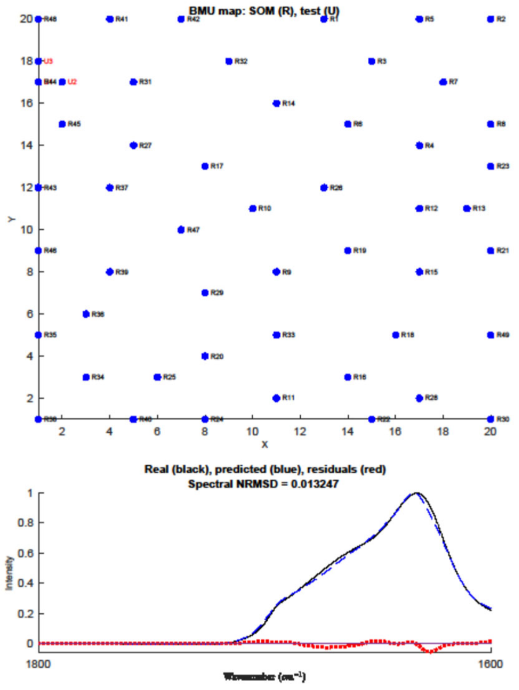

F49	F50
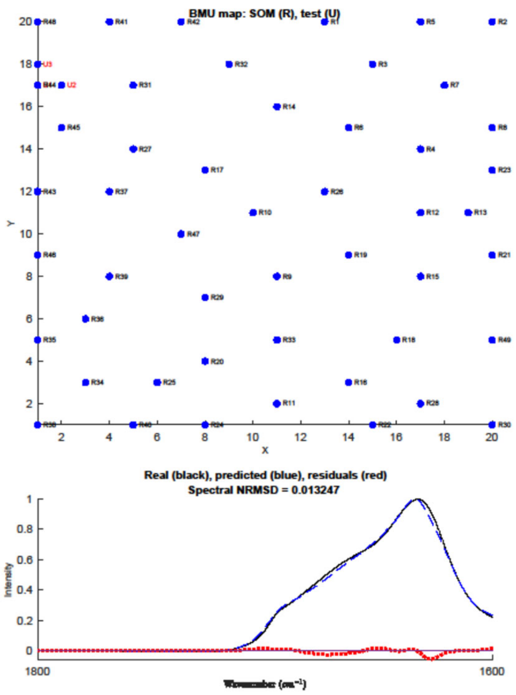	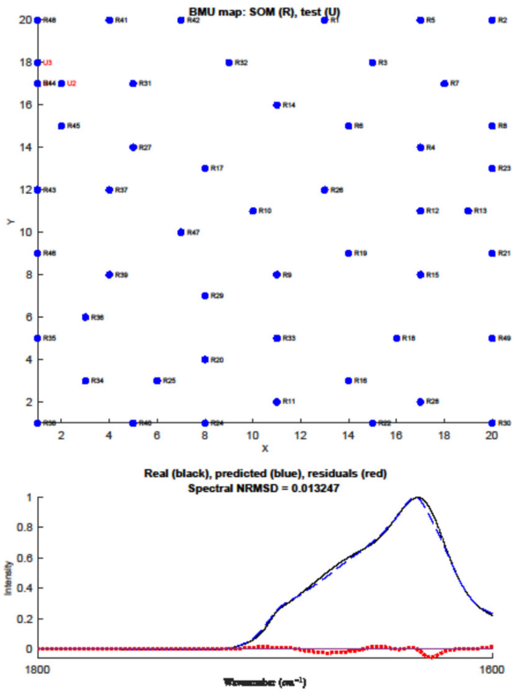

7 Direct Gaussian Band-Fitting Methodology used in this work

7.1 Origin Pro: Peak Deconvolution (Peak fitting).

Contents

1. First Fitting 19

2. Following Fittings (Method Saving) 27

3. Reporting 28

   a) Fitted Graph 28

   b) Fitted Data 29

7.2 First Fitting

Step1. Organise data and build plots (the plot peak analysis can be initiated only on the built plot)

In the data file the original data set is in the workbook “OriginalData” and plots are built in graph “AllProteins_original”

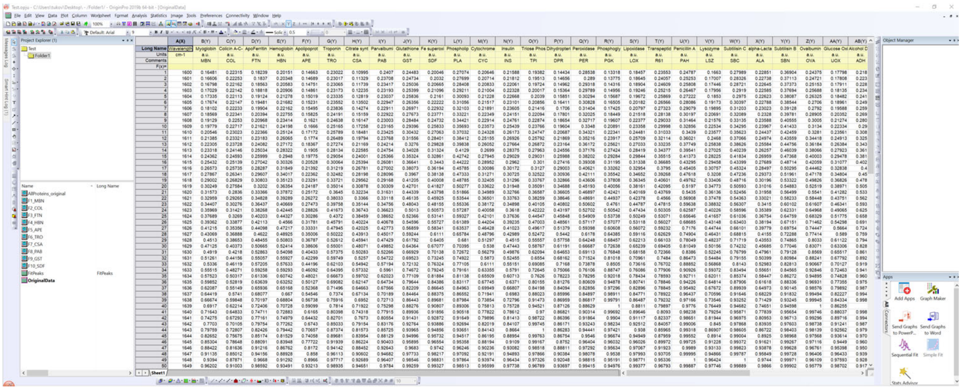

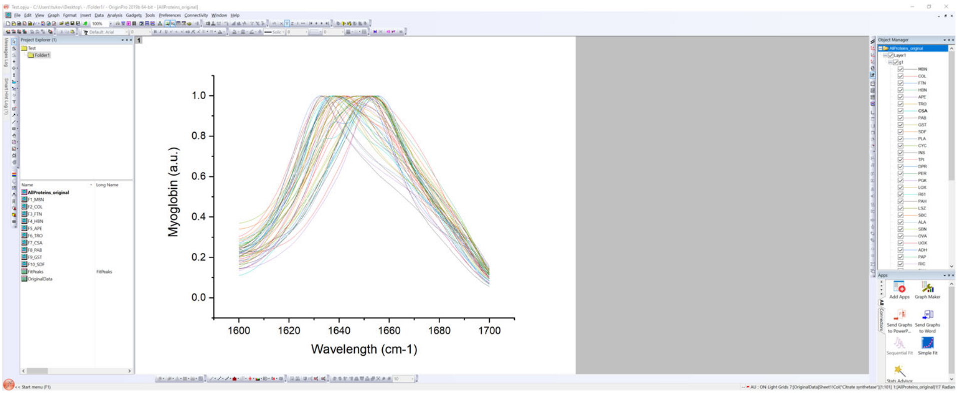

Step2. Choose the spectrum, that you want to analyse by selecting (clicking on it in the Object Manager on the right side). It will be highlighted on the graph, when selected.

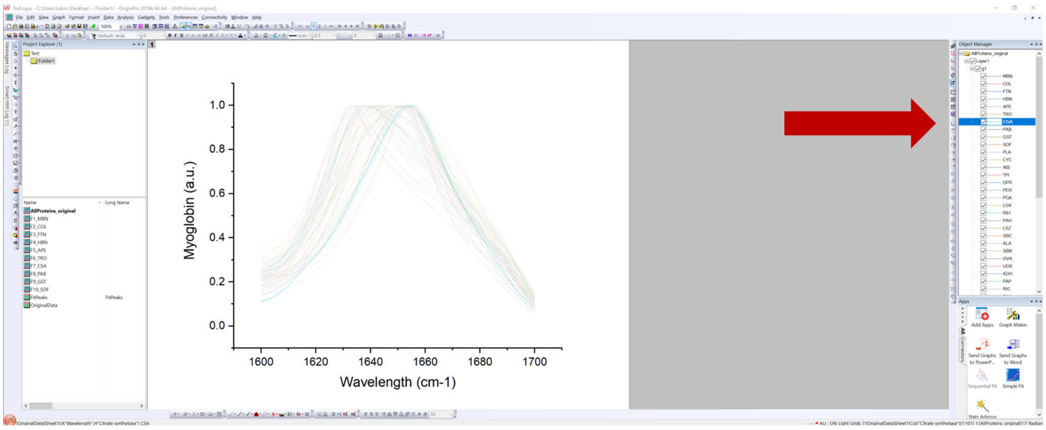

Step3. With selected graph open Analysis>Peaks and Baseline>Peal Analyzer>Open Dialog…

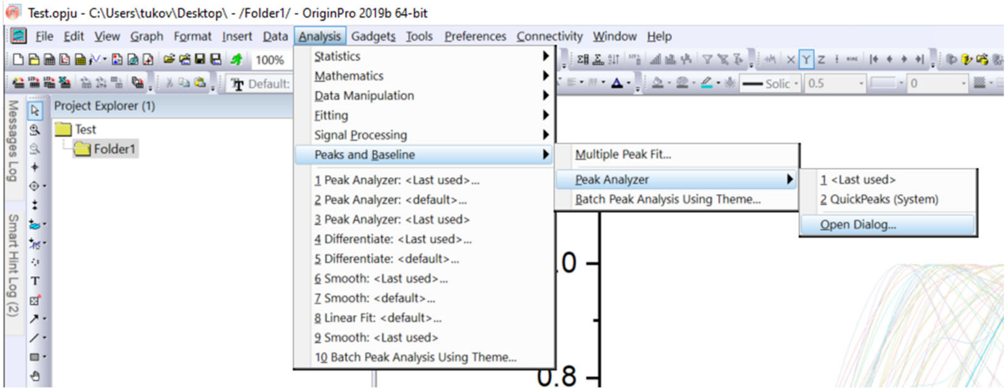

Step4. Set the goal – **Fit Peaks (Pro)** and click **Next**

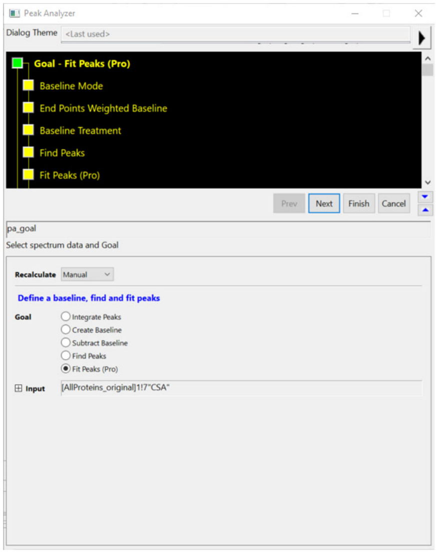

Step5. In the **Baseline Mode** choose **End Points Weighted** if the base line does not require adjustments. Click **Next**. Set **End Points(%)** to **2** and leave **Number of Baseline Points** same as input data. The end points of the graph will be connected. Click **Next.**

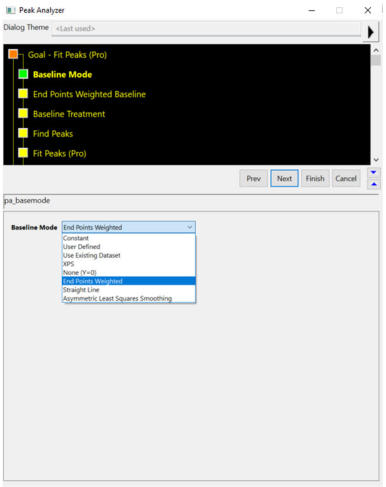

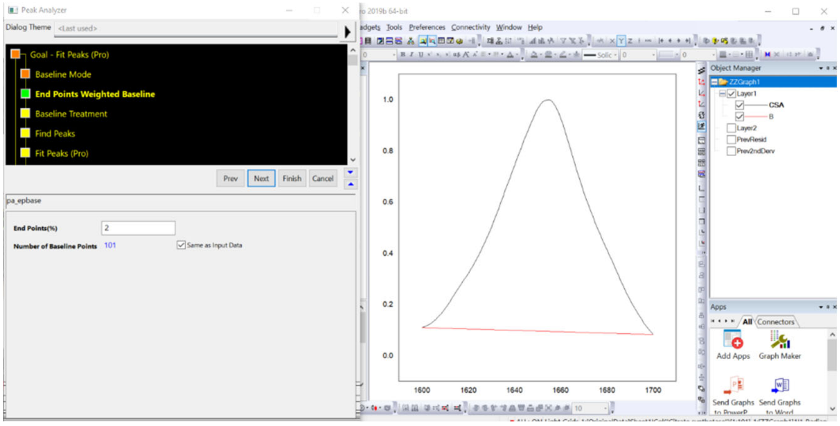

If the baseline requires adjustment, choose **User Defined** Baseline Mode> Untick **Enable Auto Find**> Click **Clear All**> Click **Add**. Choose the baseline points and click **Next**.

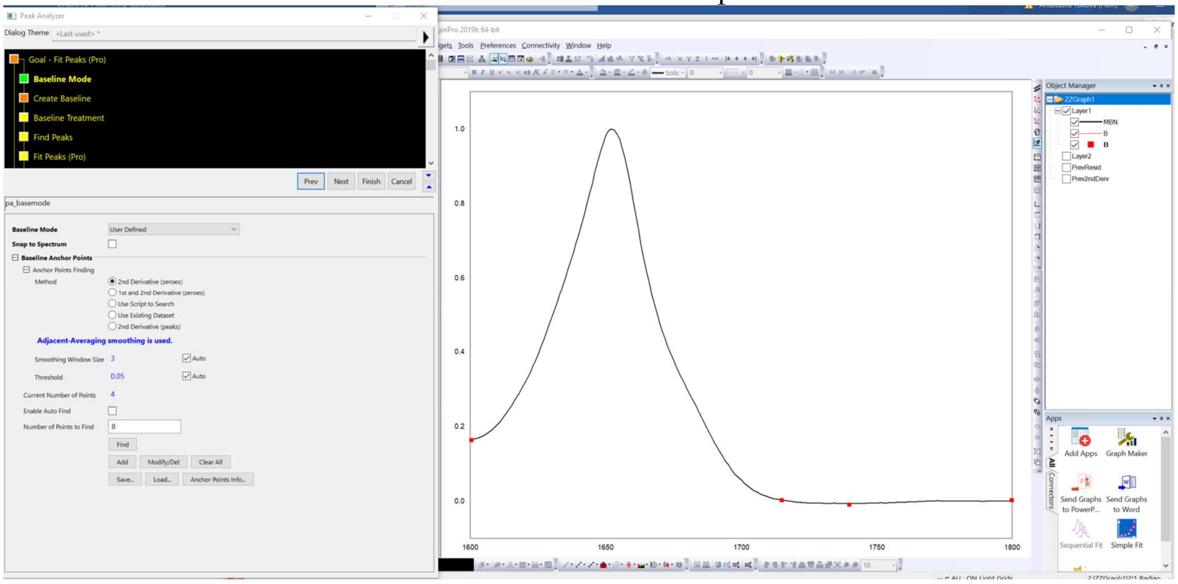

Step6. Select **Auto Subtract Baseline** and **Auto Rescale**. Click **Next**.

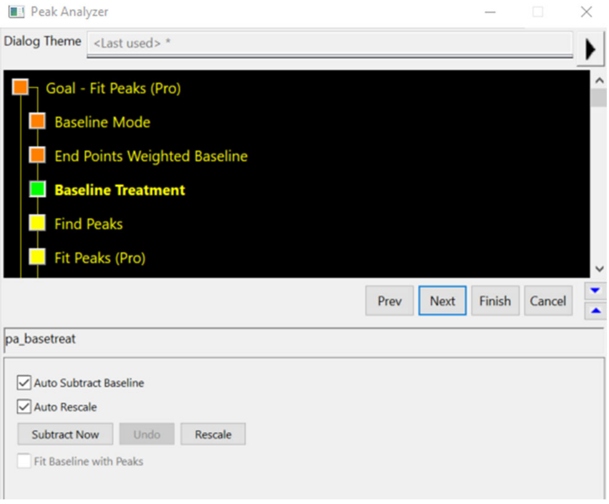

Step7. Set the Find Peaks parameter:

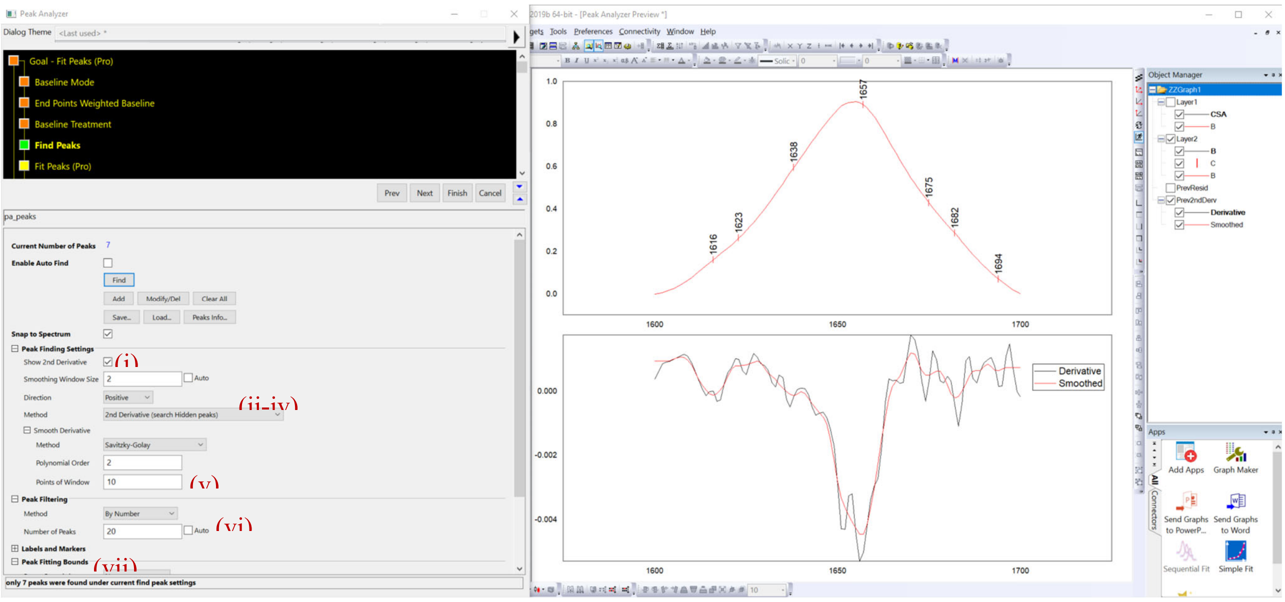

i) Go to **Peak Finding Settings**. Tick **Show 2nd Derivative.** It will show the second derivative of the analysed spectrum below it.

ii) **Smoothing Window Size** – smooths the analysed spectrum. On the above image it is marked red. Best smoothing value for the analysed spectrum is from **2** to **10 points.** The larger value may remove some important peaks. However, if the data is noisy, it is better to choose larger value, so the 2nd derivative plot is also less nosy.

iii) Set **Direction** as **Positive.** This choice excludes any peaks that fall below baseline if there is any.

iv) Choose **Method** to look for the peaks. **2nd Derivative (search hidden peaks)** was used in this example.

v) For the derivative smoothing use **Savitzky-Golay Method** with 2nd **Polynomial Order** and **Points of Window** from **7** to **10** points (the nature journal recommends 7, but I used 10 in most cases, as it reduces noise better). Make sure the smoothing doesn't move your peaks to much from original maximum (the better it matches original 2nd derivative the better fitting will be).

vi) **Peak Fitting**: Because the peaks are not very pronounces, searching by height is not efficient (it will see only the largest one or apparent maximum/minimum). Select **by Number** Method and select Number of Peaks **20**.

vii) Leave Labels and Markers and Peak Fitting Bounds unchanged, unless you want to specify that parameters.

Step8. After the parameters are set click **Find.** The number of found peaks will be displayed in the settings menu and labelled on the graph. (see image in step 7).

Step9. You can edit the number and position of found peaks if you can see some peaks, that were not detected/ you know exact position or delete peaks, that are not relevant. It will improve fitting. You can do it in the **Find Peaks** window or click Next and do it in the **Fit Peaks** window.

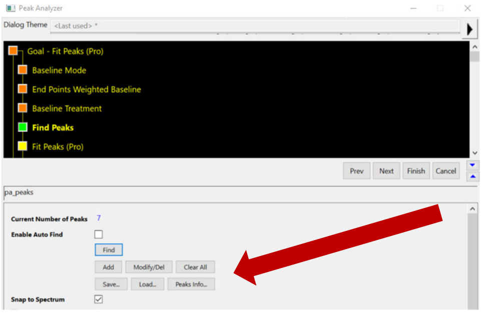

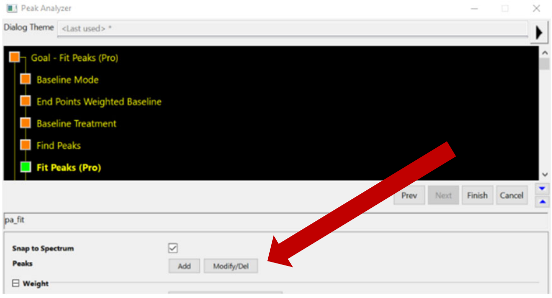

Step10. When the peaks are determined click **Fit Control**.

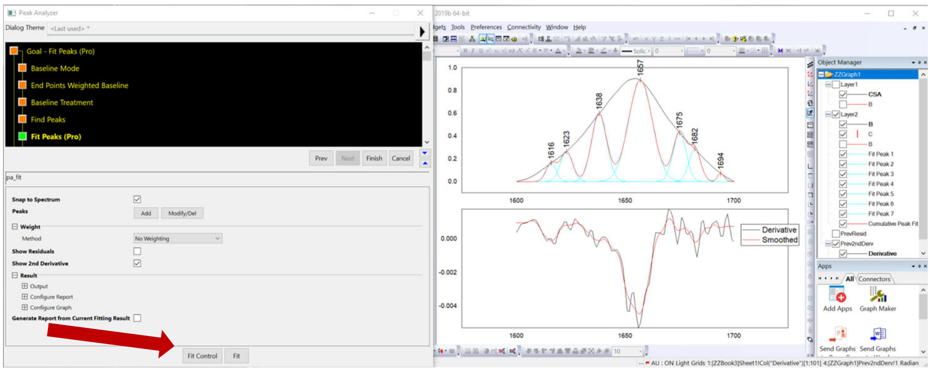

Step11. In the Fit Control window:

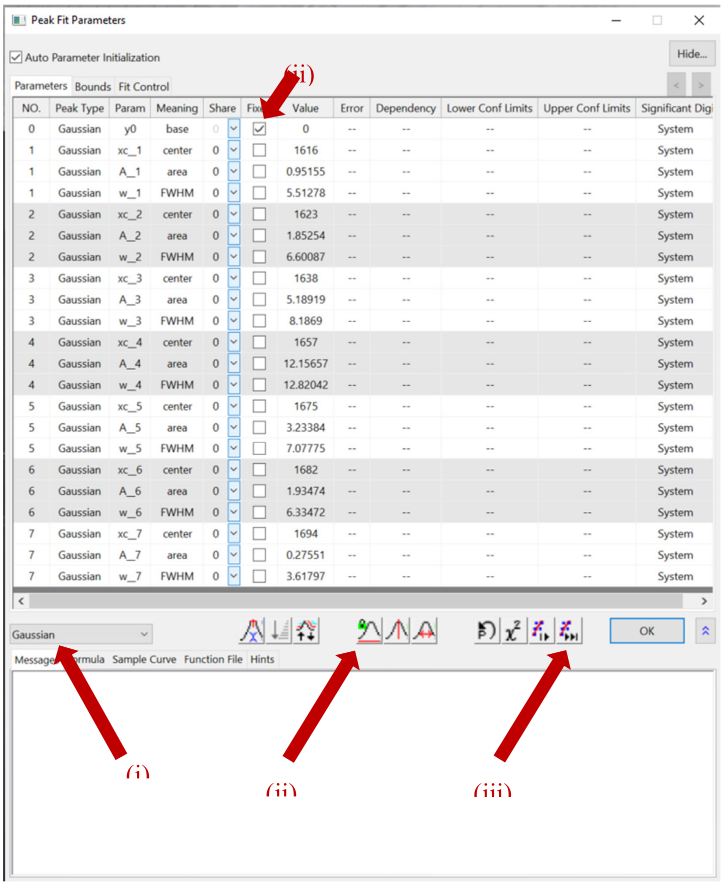

i) Use Gaussian for the fitting.

ii) Click Fix baseline and make sure the there is a tick in the baseline Parameter window.

ii) Click Fit until converged.

Sep12. The program will do the fitting and present the parameters in Message Window.

i)You can visually determine if the fitting was satisfactory as well as checking COD(R^2) number in the message window (It should be as close to 1 as possible, I considered good fitting with values > 0.999 and satisfactory with 0.999>x>0.990).

If the fitting is bad you can:

ii) Try to change second derivative plot (change smoothness, fix peak positions), so the peaks are updated (you will need to close the Fit Control window and go to previous window where you've set peak find parameters (Step7).

iii) Try to change Bounds if the fitted peaks look too off (for example thin and tall like a line or tiny bump). I've used bound change only in 3 cases PGK, OVA and RNA plots, because other manipulations didn't work. But it is always better to fix derivative plot first.

iv) If the fitting is only a little bit off, try to do multiple iterations. By default, the program counts to 200, the maximum for one run is 500 – it can be set in **Fit Control** tab, but you can click **Fit until**

converged multiple times to double iterations for better fit. This works only when a small correction needed.

v) Click OK after fitting is done.

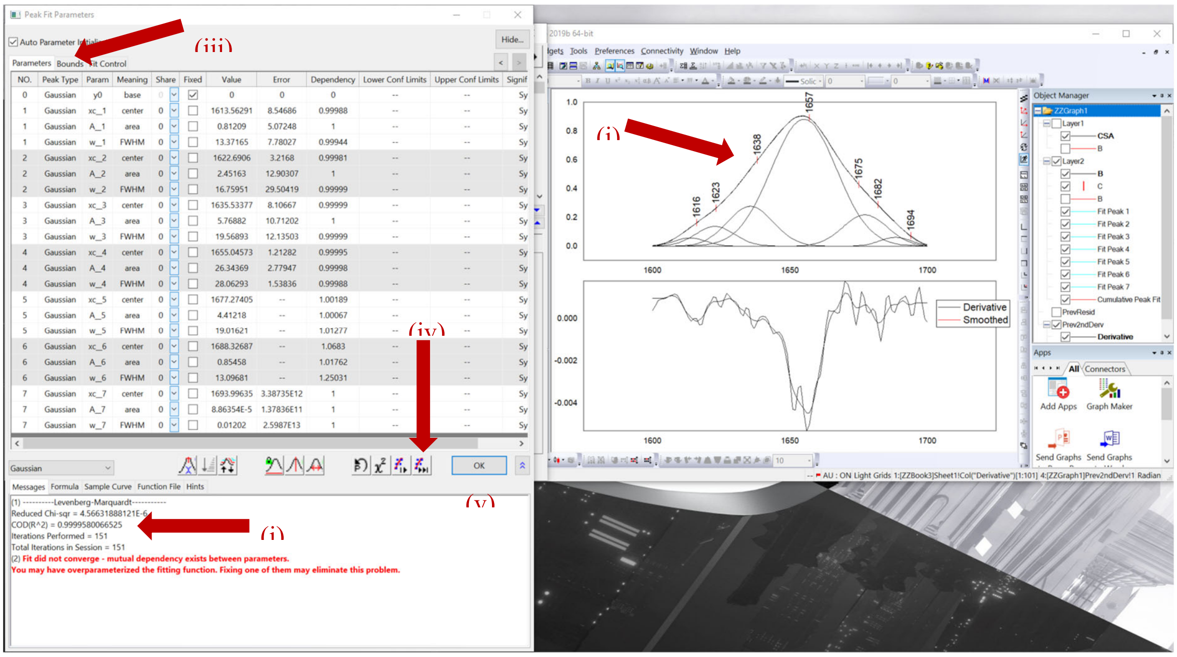

Step13. Before finishing peak analysis set up report parameters: In **Result** section open **Output**. Here you can set where your results will be stored (new workbook, name of data files and so on), what data you want to see in report tables, graphs.

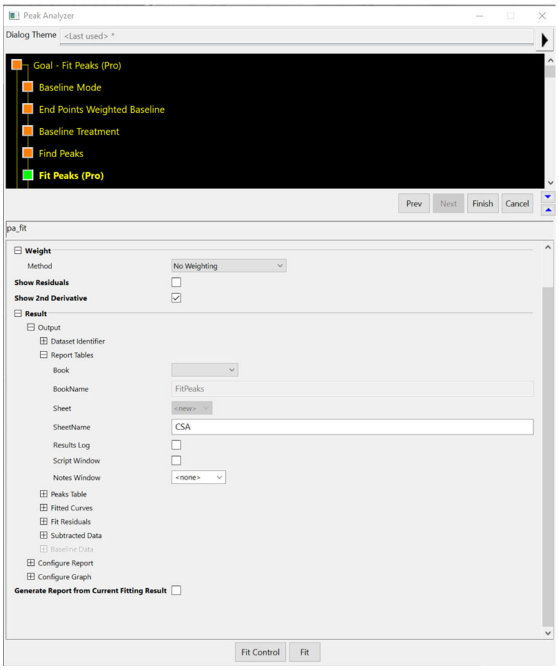

Step 14. Click **Finish**. Your data report will be generated.

7.3 Following Fittings (Method Saving)

Step1. The next fitting can be done using same parameters. For that save the fitting parameters. Before clicking Finish (Step14 in First Fitting chapter). Select black arrow> Save As…

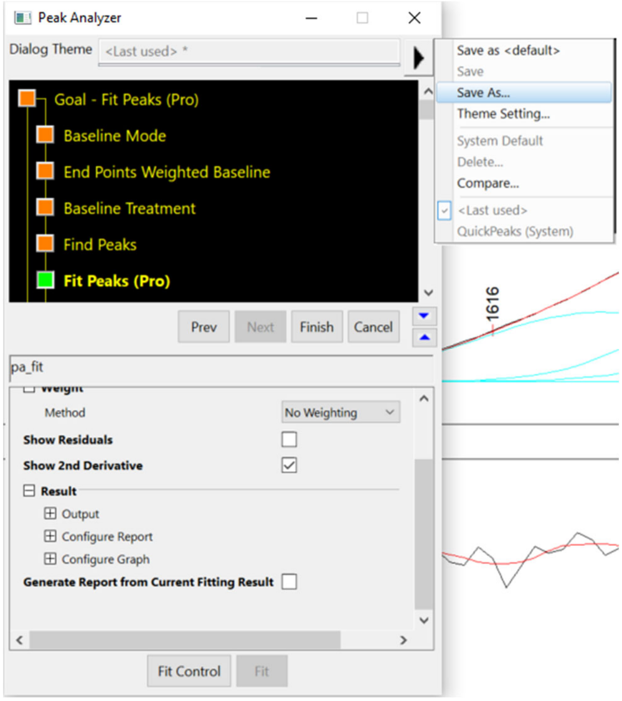

Step2. Specify name and fitting options, that you want to repeat. Click OK.

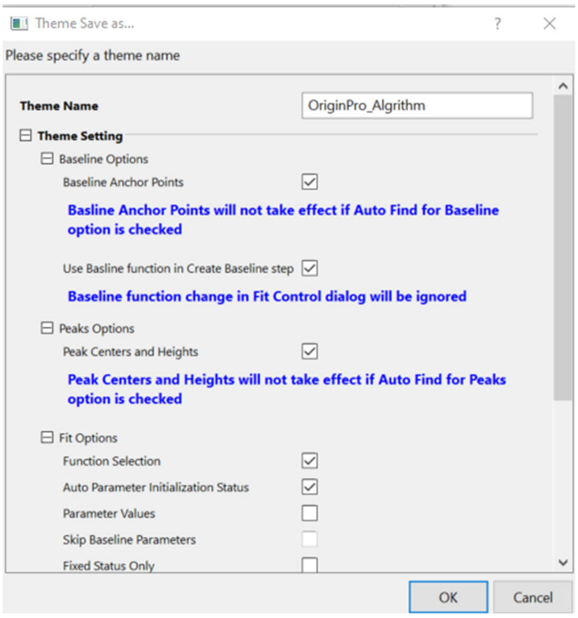

Step3. You can call the algorithm by selecting it in the previously used record list or in the Peak Analyzer tab. But, it will automatically process your selected data and give you result! There will be no option to change any of the parameters. (Mostly it is useless, because you need to adjust fitting parameters all the time, but might be used in some cases).

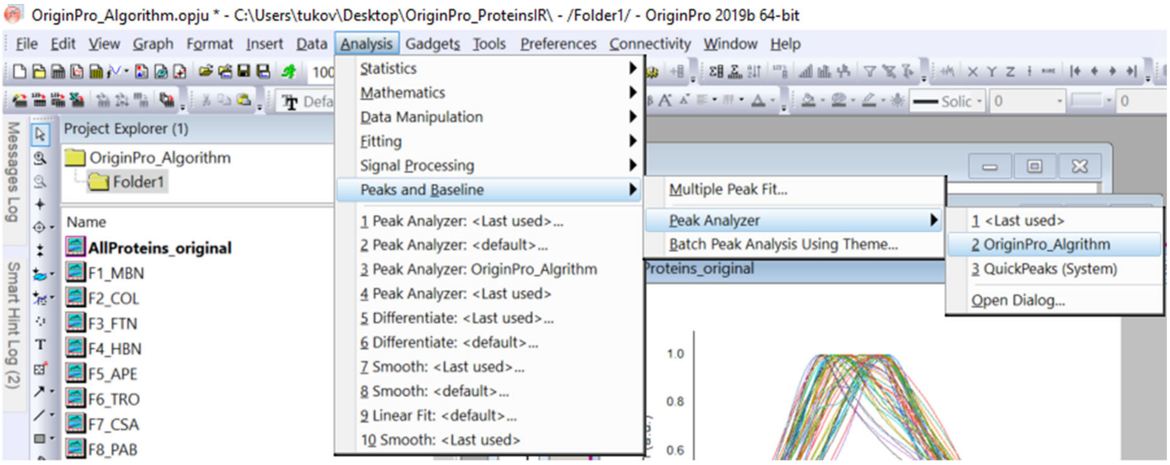

The better way is to go to Analysis>Peaks and Baseline>Peak Analyser>Open Dialog…

In opened dialog window, click on black arrow and select your saved method. That way, you’ll scroll through the same windows, but the parameters of the algorithm will already be filled in, you only adjust where needed.

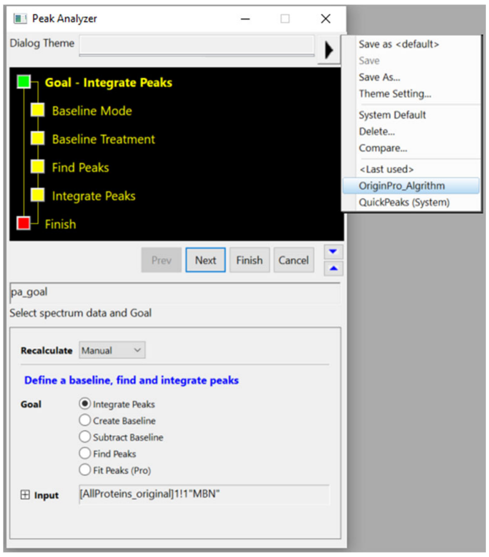

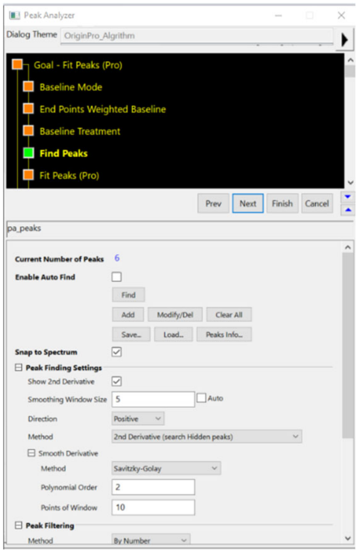

7.4 Reporting

The processed data is saved as:

7.4.1 a) Fitted Graph

1. All graphs are labelled with their F number from original dataset and their short name in the Project Explorer window.

2. The Peak Analysis graph has short summary of fitting parameters.

3. Graph lines are labelled in the In the Object Manager window. The fitted peaks can be visually analysed here (how relevant they are, and removed from data set if required).

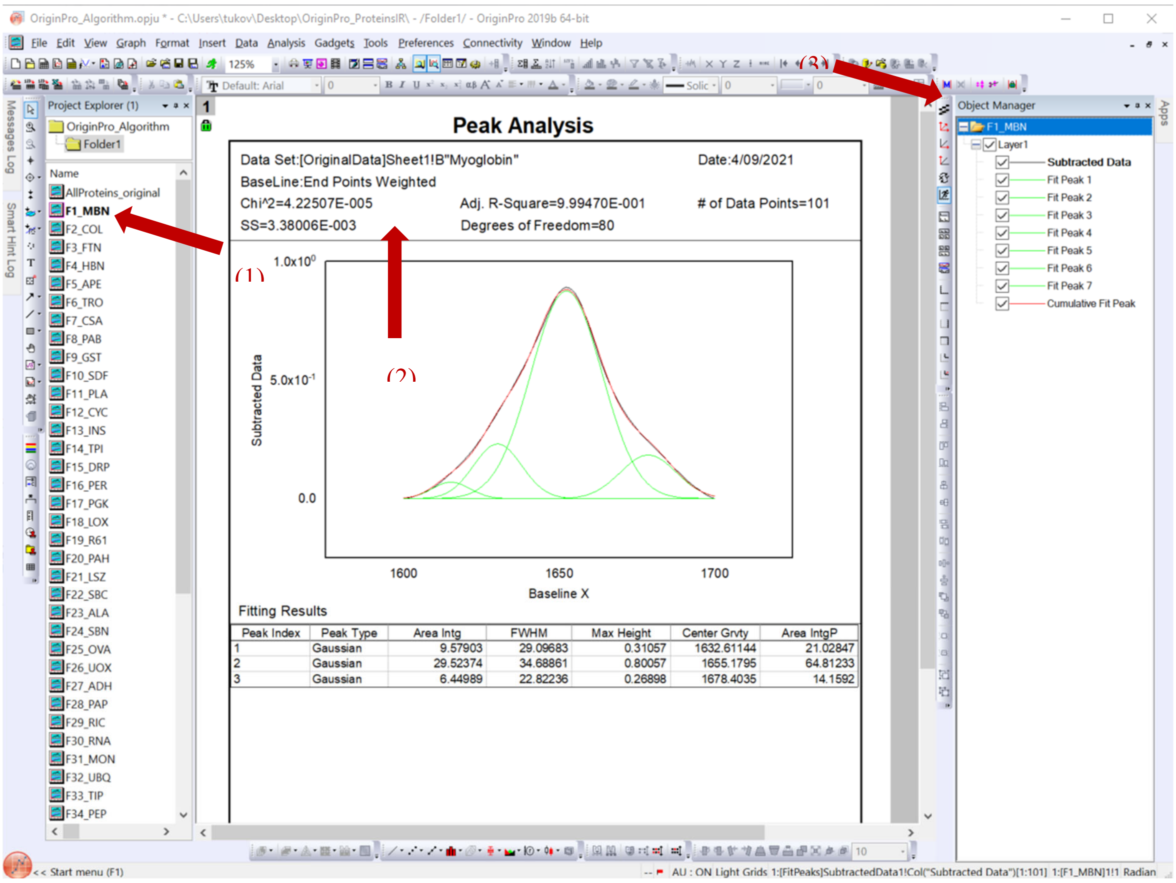

7.4.2 b) Fitted Data

1. The fitted data is saved in a separate workbook “FitPeaks”.
2. Each protein record has fitting report labelled as protein’s abbreviation and three separate sheets:
i) FitPeakCurve – data points that are used to build Fitted Graph;
ii) PeakProperties – has data about fitted peaks’ area, height etc.;
ii) SubtractedData – baseline data points.

8 Origin Pro: Peak Deconvolution (Peak fitting).

Contents

1. First Fitting 19

2. Following Fittings (Method Saving) 27

3. Reporting 28

a) Fitted Graph 28
b) Fitted Data 29

8.1 First Fitting

Step1. Take the second derivative of each data set (unfortunately have to do it separately for each protein). Select the whole column> Analysis> Mathematics>Differentiate>Open Dialog

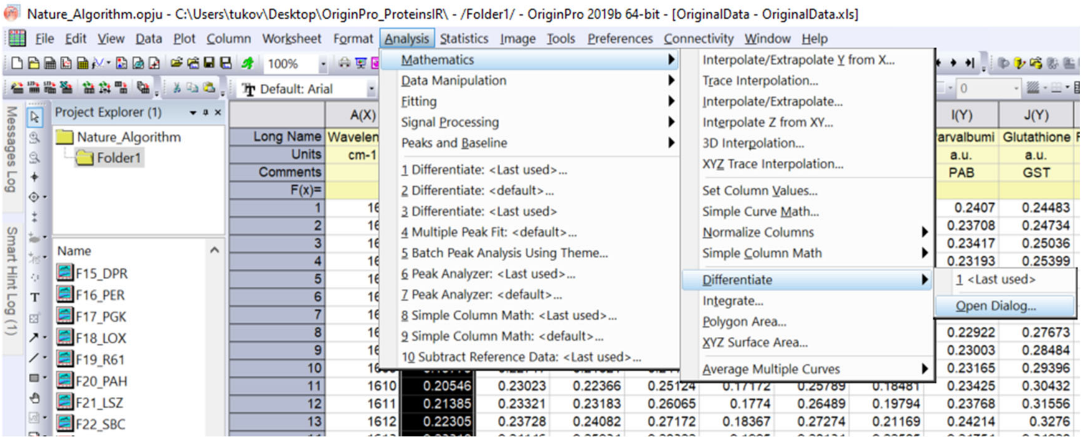

In the dialog window, set the parameters an on the image below. Don’t change output directory (the data will be saved in the same workbook and same sheet (right after the original data)

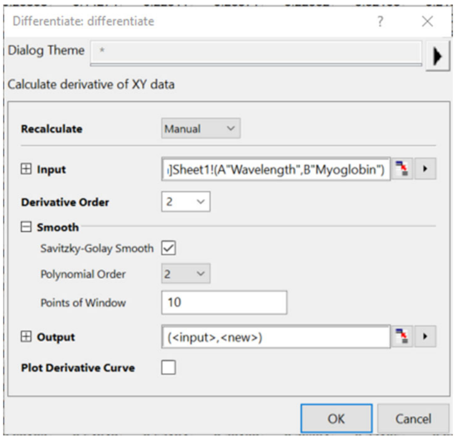

Step3. After all derivatives are ready build all of them as plots.

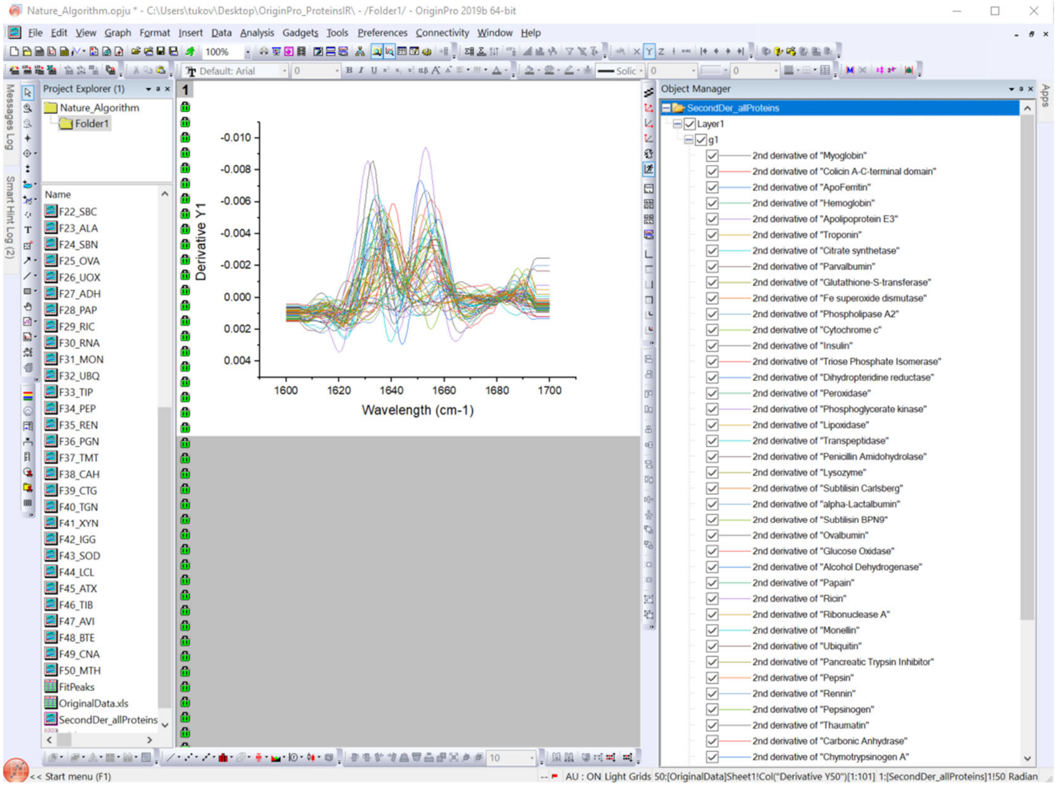

Step4. Select one plot, that you want to analyse (click on its name in the object manager)then click Analysis> Peaks and Baseline > Peak Analyzer > Open dialog

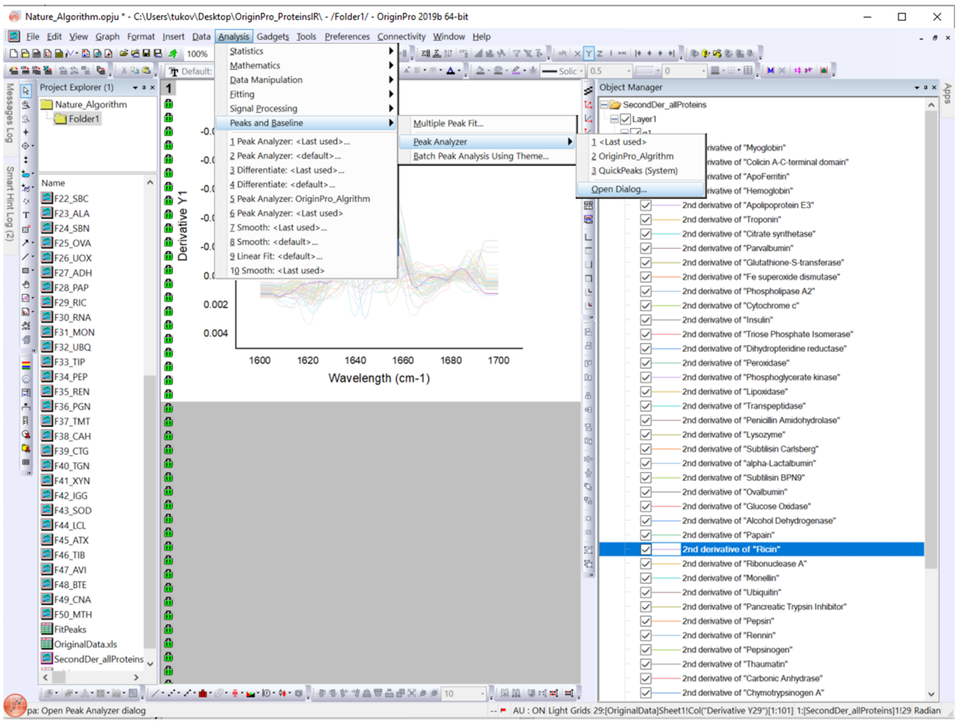

Step5. In the opened window select Fit Peaks (Pro)> Next

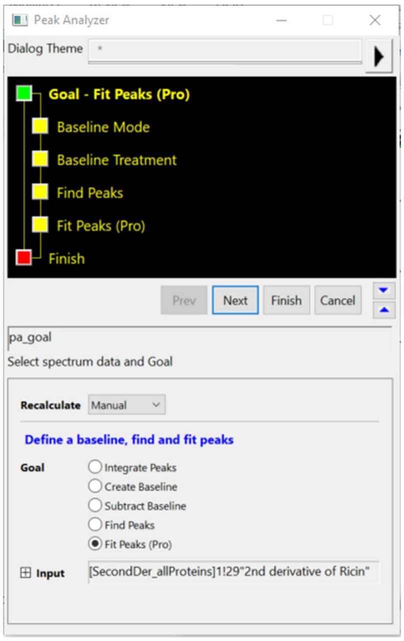

Step6. Select User Defined Baseline Mode. Untick Enable Auto Find> Click Clear All> Click Add

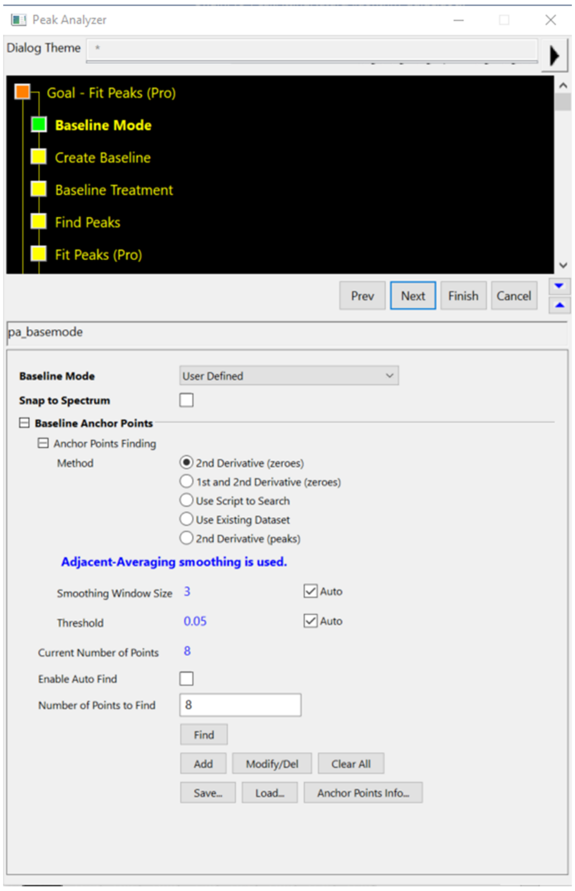

Step6. Manually choose baseline points. Unfortunately this is the step that can’t be automated. Select the points of minimums on the graph, that go through the main baseline connecting right and left side. Click Next.

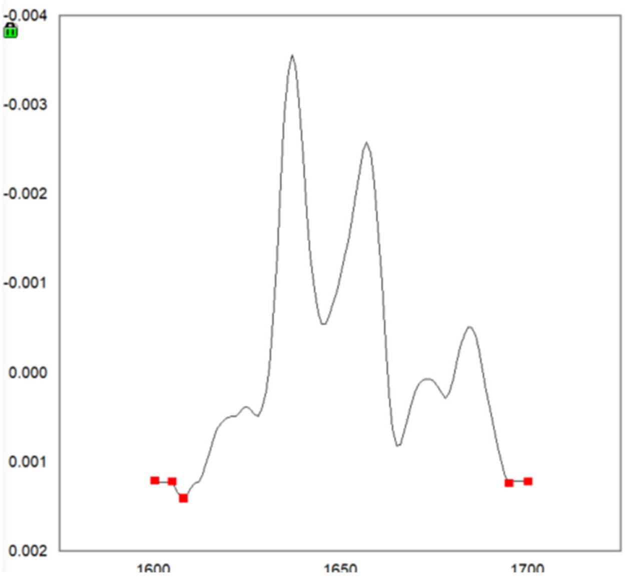

Step 7. The software connects the points so you can see where the baseline will be. You can adjust the points by clicking Add of Modify/Del.

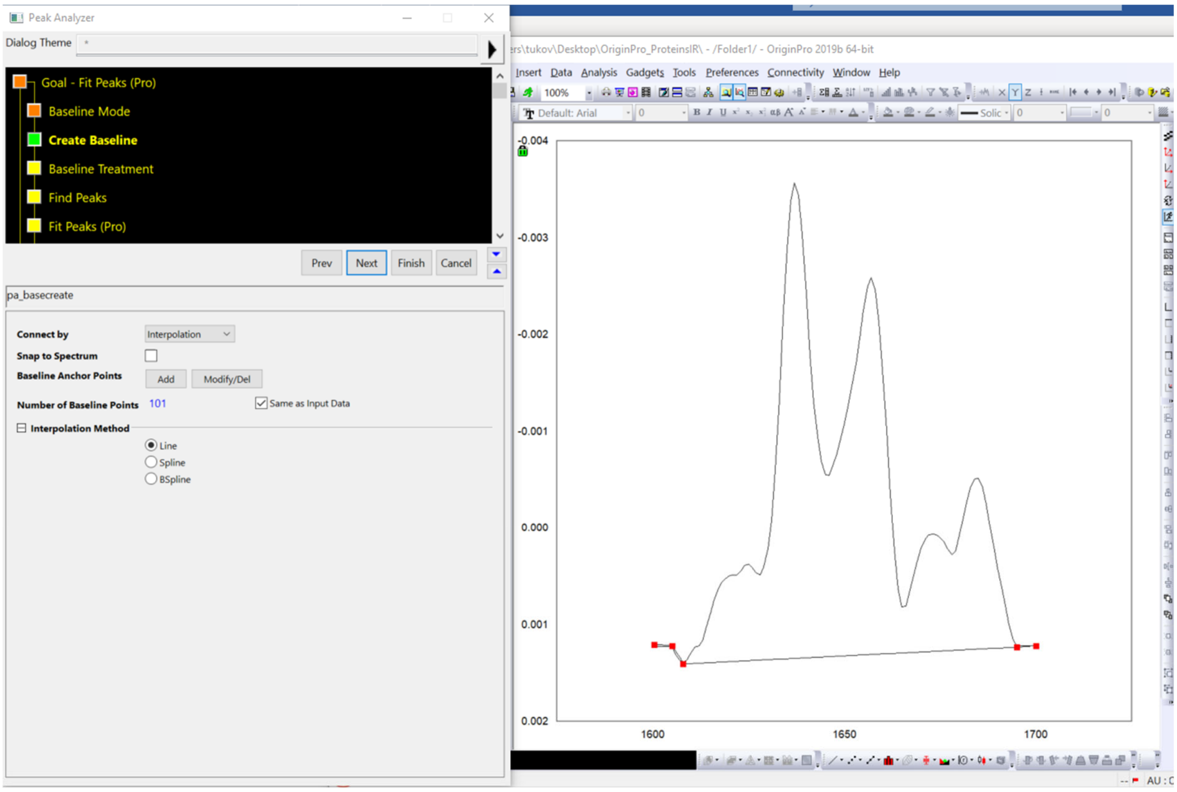

Step8. Tick auto Subtract Baseline and Auto Rescale. Click Next.

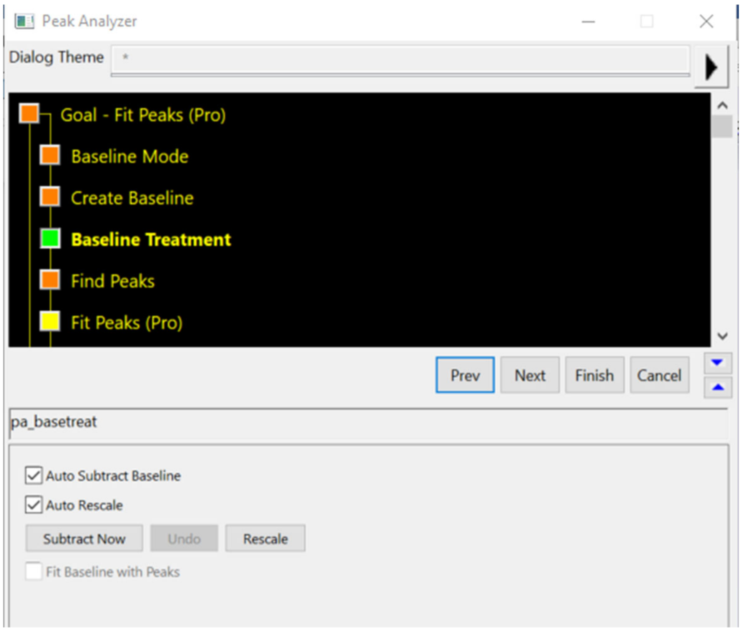

Step9. i) Untick Enable Auto Find
ii) In Peak Finding Settings select Smoothing Window Size 5 (The larger smoothing moves peaks positions too much), set Direction Negative (it will look only for negative peaks) and use Local Maximum Method to look for peaks. Set Local Points as 2.
iii) Set Peak Filtering by Number with 20 peaks.
iv) Leave Labels and Markers and Peak Fitting Bounds Unchanged, unless you want to specify them.
v)Click Find

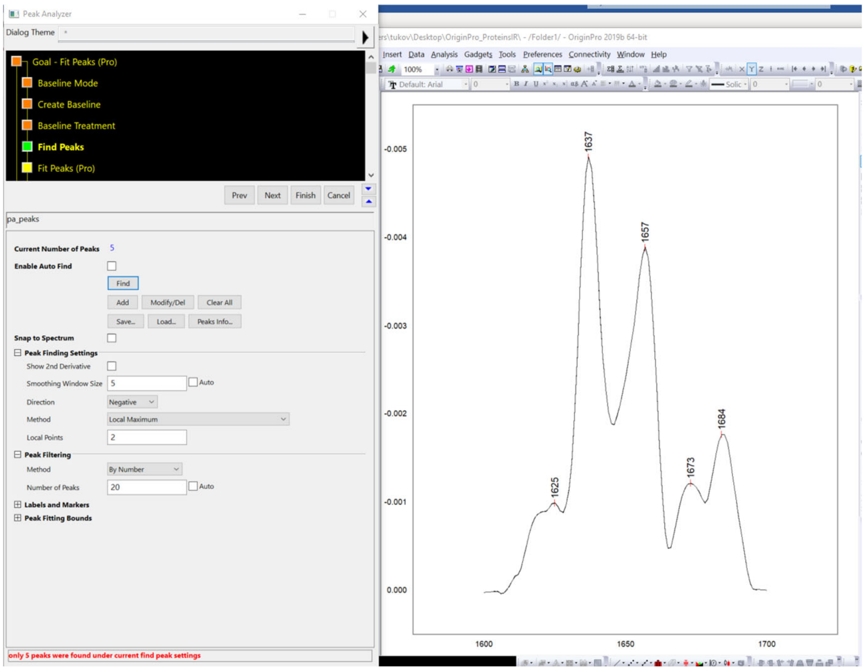

Step 10. At this point, the program will find the most apparent peaks. However, you'll need to add more peaks (that were not picked by program) manually. This is very important to set all possible peaks for the fitting.

You can edit the number and position of found peaks if you can see some peaks, that were not detected/ you know exact position or delete peaks, that are not relevant. You can do it in the **Find Peaks** window or click Next and do it in the **Fit Peaks** window.

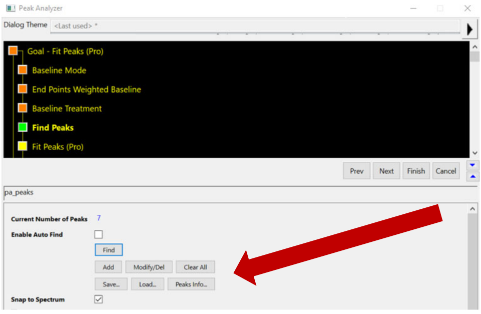

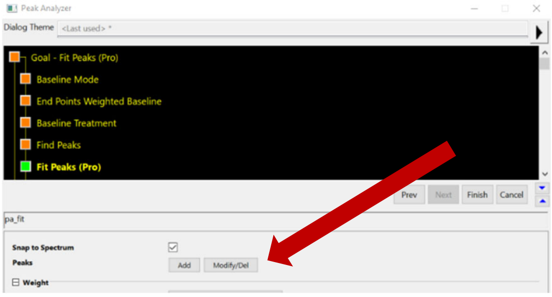

When the peaks are determined click **Fit Control**.

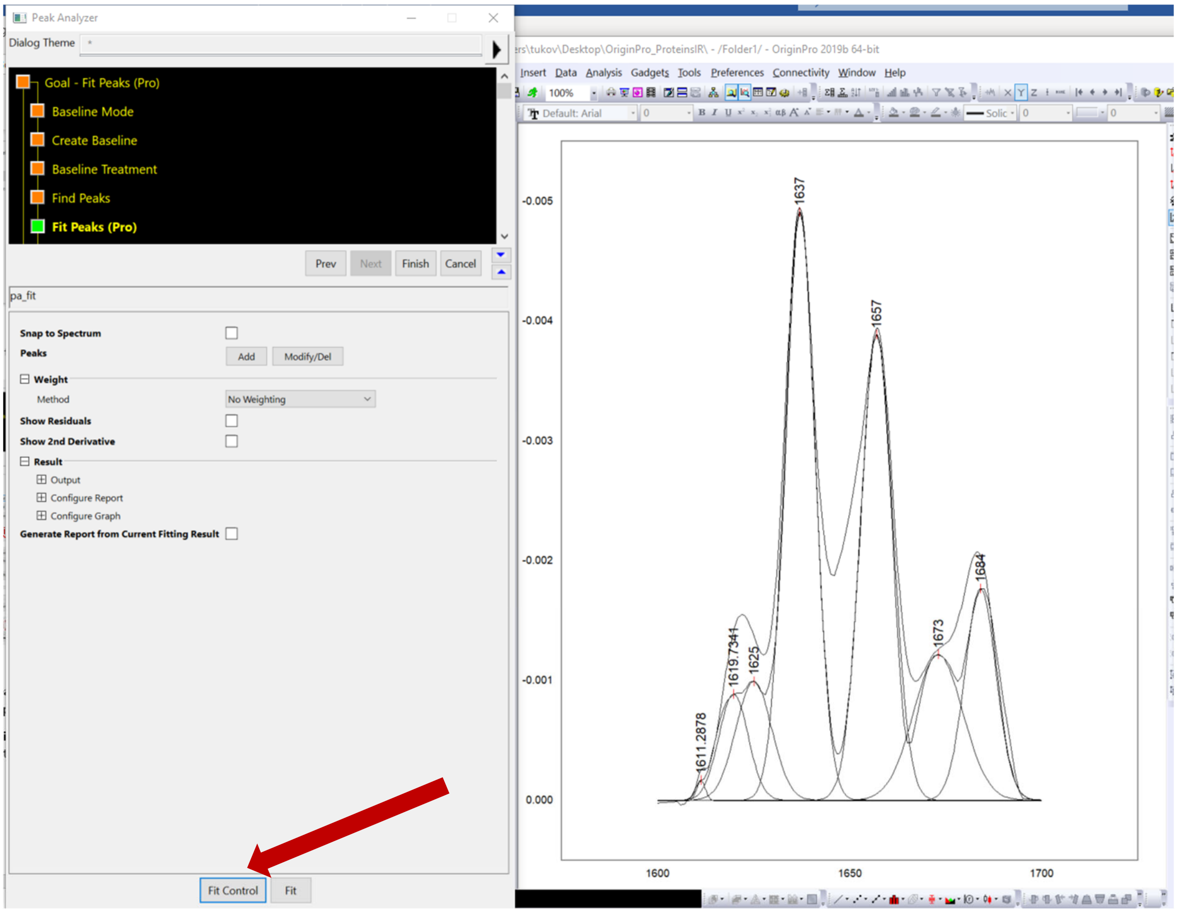

Step11. In the Fit Control window:
i) Use Gaussian for the fitting.
ii) Click Fix baseline and make sure the there is a tick in the baseline Parameter window.
ii) Click Fit until converged.

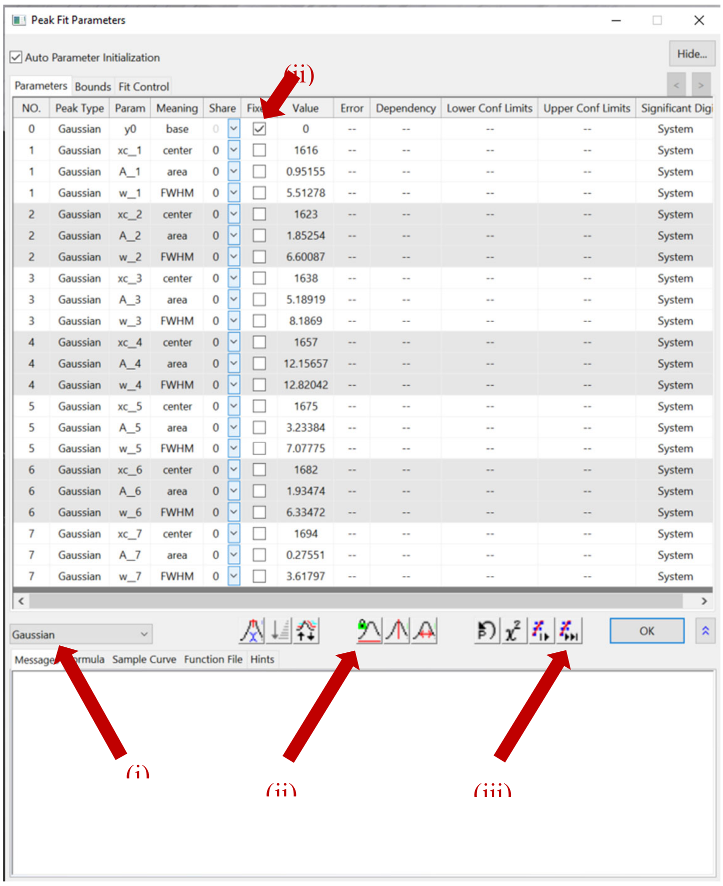

Sep12. The program will do the fitting and present the parameters in Message Window.

i)You can visually determine if the fitting was satisfactory as well as checking COD(R^2) number in the message window (It should be as close to 1 as possible, I considered good fitting with values > 0.999 and satisfactory with 0.999>x>0.990).

If the fitting is bad you can:

ii) Try to detect more peaks. It is usually the best way to fix fitting. If the plot's peak is too broad, have a slight shoulder, try to approximately guess, where the peak might be and add a point (Step10). The program will adjust peaks positions for the fitting anyway, so don't worry about precise position of the "guess" peak.

iii) Try to change Bounds if the fitted peaks look too off (for example thin and tall like a line or tiny bump

iv) If the fitting is only a little bit off, try to do multiple iterations. By default, the program counts to 200, the maximum for one run is 500 – it can be set in **Fit Control** tab, but you can click **Fit until converged** multiple times to double iterations for better fit. This works only when a small correction needed.

v) Click OK after fitting is done.

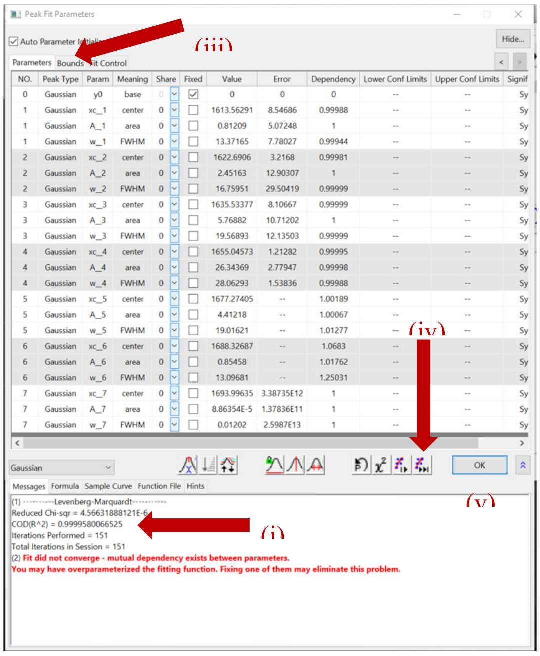

Step13. Before finishing peak analysis set up report parameters: In **Result** section open **Output**. Here you can set where your results will be stored (new workbook, name of data files and so on), what data you want to see in report tables, graphs.

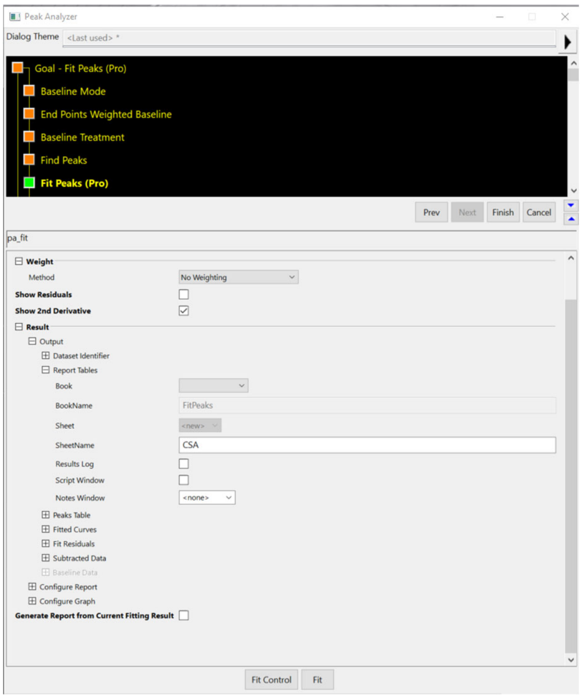

Step 14. Click **Finish**. Your data report will be generated.

8.2 Following Fittings (Method Saving)

Step1. The next fitting can be done using same parameters. For that save the fitting parameters. Before clicking Finish (Step14 in First Fitting chapter). Select black arrow> Save As…

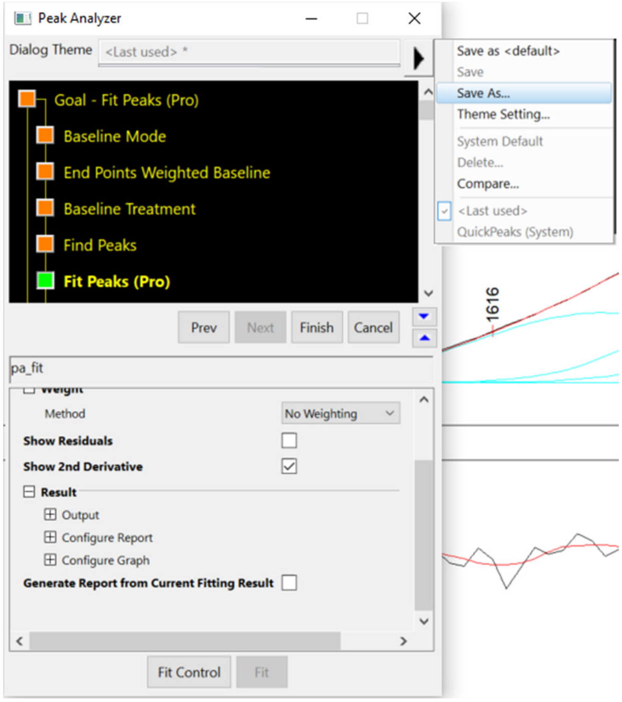

Step2. Specify name and fitting options, that you want to repeat. Click OK.

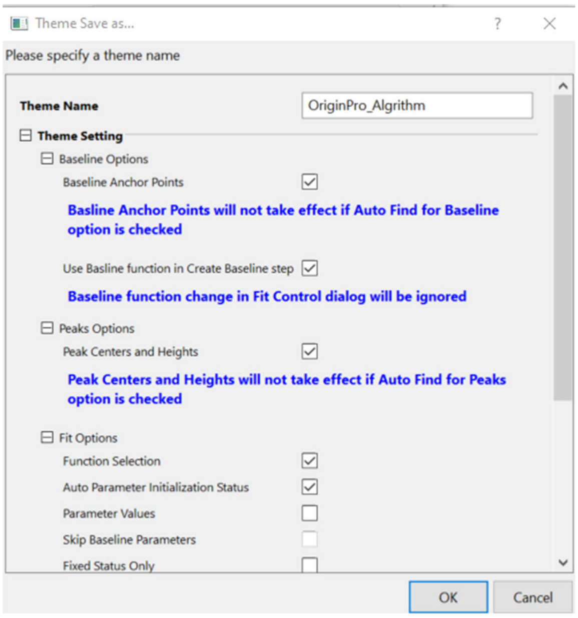

Step3. You can call the algorithm by selecting it in the previously used record list or in the Peak Analyzer tab. But, it will automatically process your selected data and give you result! There will be

no option to change any of the parameters. (Mostly it is useless, because you need to adjust fitting parameters all the time, but might be used in some cases).

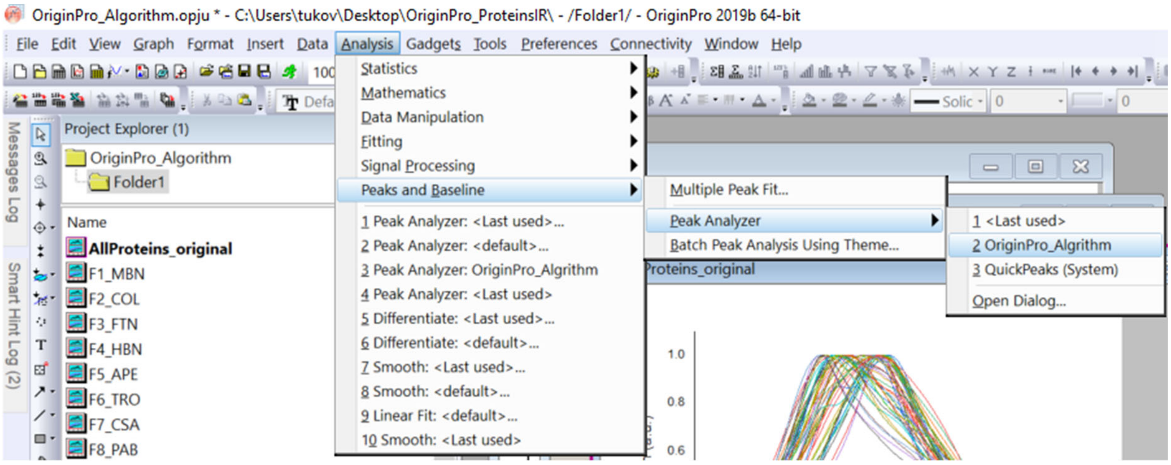

The better way is to go to Analysis>Peaks and Baseline>Peak Analyser>Open Dialog…

In opened dialog window, click on black arrow and select your saved method. That way, you'll scroll through the same windows, but the parameters of the algorithm will already be filled in, you only adjust where needed.

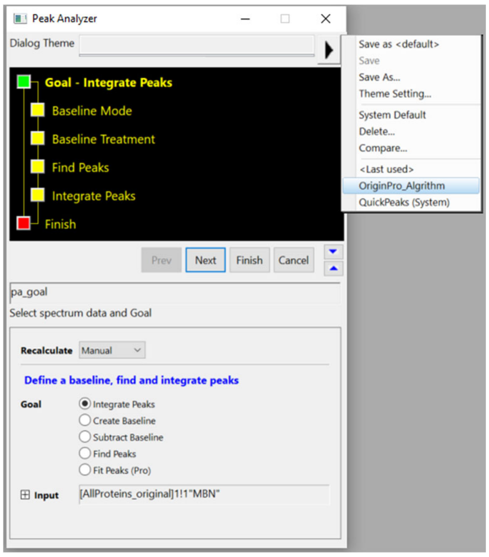

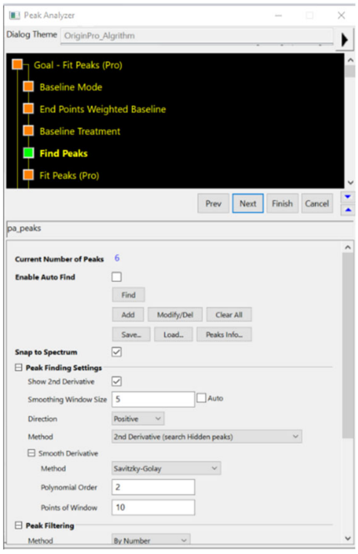

8.3 Reporting

The processed data is saved as:

8.3.1 a) Fitted Graph

1. All graphs are labelled with their F number from original dataset and their short name in the Project Explorer window.

2. The Peak Analysis graph has short summary of fitting parameters.

3. Graph lines are labelled in the In the Object Manager window. The fitted peaks can be visually analysed here (how relevant they are, and removed from data set if required).

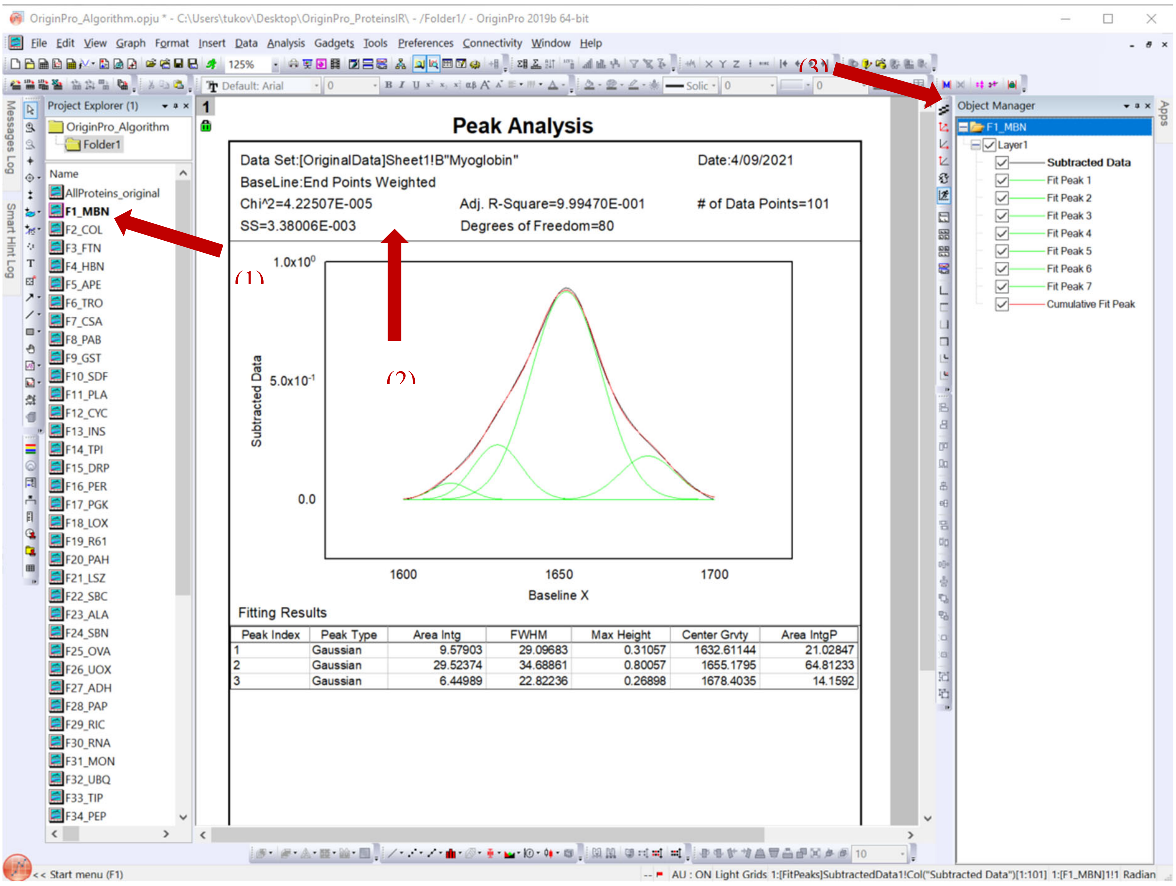

8.3.2 b) Fitted Data

1. The fitted data is saved in a separate workbook “FitPeaks”.

2. Each protein record has fitting report labelled as protein’s abbreviation and three separate sheets:

i) FitPeakCurve – data points that are used to build Fitted Graph;

ii) PeakProperties – has data about fitted peaks’ area, height etc.;

ii) SubtractedData – baseline data points.

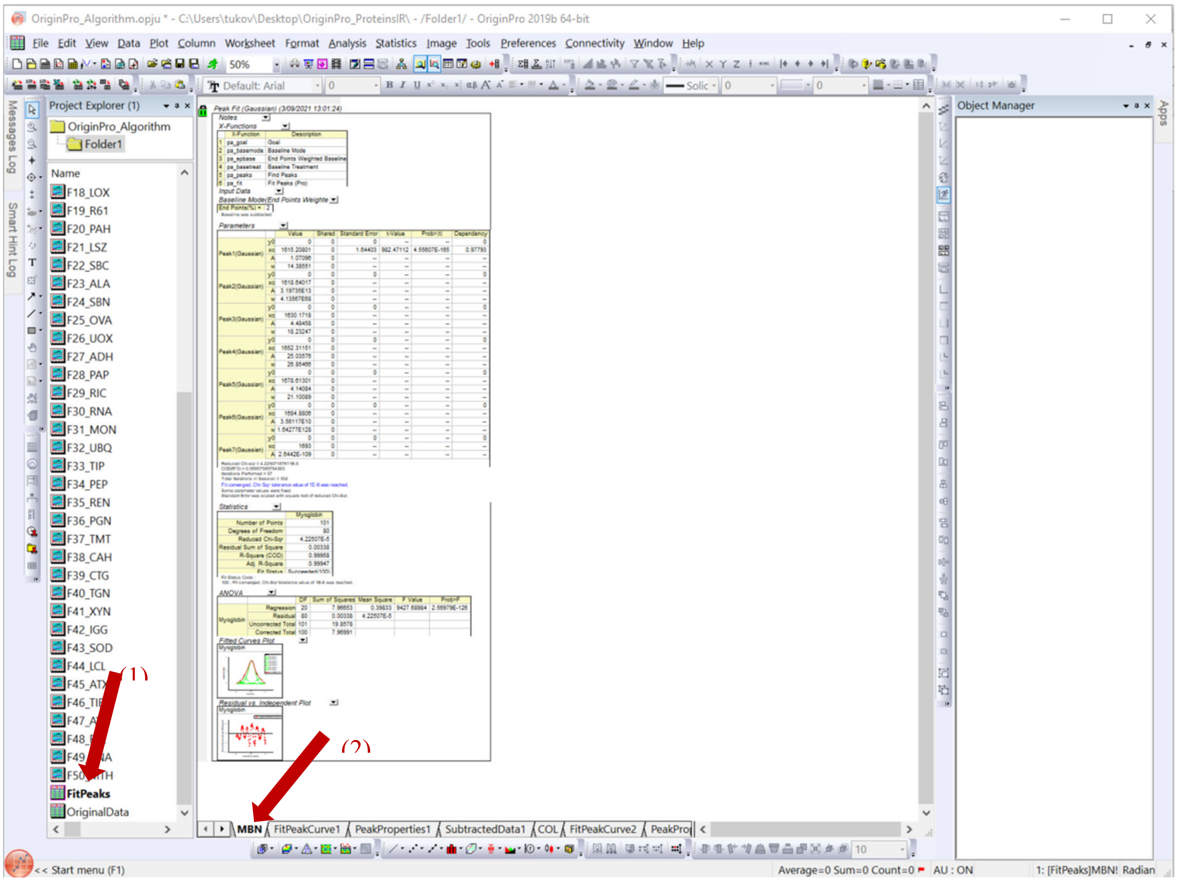

Hint: for fast orientation right click with mouse on the white space in the report and select Show Organiser. It will open the window, where you can select report or data sheet you want to check.

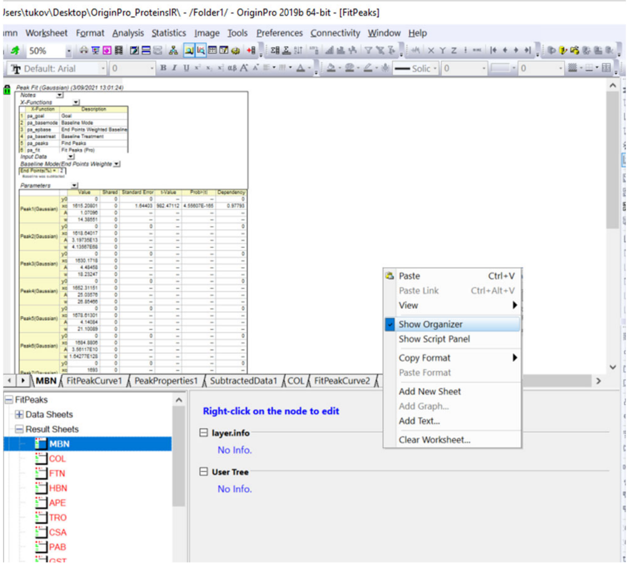

9 References

Corujo, M.P., Sklepari, M., Ang, D.L., Millichip, M., Reason, A., Goodchild, S.C., Wormell, P., Amarasinghe, D.P., Lindo, V., Chmel, N.P., and Rodger, A. (2018). Infrared absorbance spectroscopy of aqueous proteins: Comparison of transmission and ATR data collection and analysis for secondary structure fitting. *Chirality* 30**,** 957-965.

Goormaghtigh, E., Ruysschaert, J.-M., and Raussens, V. (2006). Evaluation of the Information Content in Infrared Spectra for Protein Secondary Structure Determination. *Biophysical Journal* 90**,** 2946-2957.

Hall, V., Nash, A., Hines, E., and Rodger, A. (2013). Elucidating protein secondary structure with circular dichroism and a neural network. *J. Comp. Chem.* 34**,** 2774–2786.

Hall, V., Nash, A., and Rodger, A. (2014a). SSNN, a method for neural network protein secondary structure fitting using circular dichroism data. *Analytical Methods* 6**,** 6721-6726.

Hall, V., Sklepari, M., and Rodger, A. (2014b). Protein secondary structure prediction from circular dichroism spectra using a self-organizing map with concentration correction. *Chirality* 26**,** 471-482.

Kohonen, T. (1982). Self-organized formation of topologically correct feature maps. *Biological Cybernetics* 43**,** 59-69.

Rodger, A., Steel, M.J., Goodchild, S.C., Chmel, N.P., and Reason, A. (2020). Transformation of aqueous protein attenuated total reflectance infra-red absorbance spectroscopy to transmission. *QRB Discovery* 1**,** e8.

Yang, H., Yang, S., Kong, J., Dong, A., and Yu, S. (2015). Obtaining information about protein secondary structures in aqueous solution using Fourier transform IR spectroscopy. *Nature Protocols* 10**,** 382.
